# Applying of Hierarchical Clustering to Analysis of Protein Patterns in the Human Cancer-Associated Liver

**DOI:** 10.1371/journal.pone.0103950

**Published:** 2014-08-01

**Authors:** Natalia A. Petushkova, Mikhail A. Pyatnitskiy, Vladislav A. Rudenko, Olesya V. Larina, Oxana P. Trifonova, Julya S. Kisrieva, Natalia F. Samenkova, Galina P. Kuznetsova, Irina I. Karuzina, Andrey V. Lisitsa

**Affiliations:** 1 V. N. Orekhovich Institute of Biomedical Chemistry, Russian Academy of Medical Sciences, Moscow, Russia; 2 Postgen Tech LLC, Moscow, Russia; Università della Calabria, Italy

## Abstract

**Background:**

There are two ways that statistical methods can learn from biomedical data. One way is to learn classifiers to identify diseases and to predict outcomes using the training dataset with established diagnosis for each sample. When the training dataset is not available the task can be to mine for presence of meaningful groups (clusters) of samples and to explore underlying data structure (unsupervised learning).

**Results:**

We investigated the proteomic profiles of the cytosolic fraction of human liver samples using two-dimensional electrophoresis (2DE). Samples were resected upon surgical treatment of hepatic metastases in colorectal cancer. Unsupervised hierarchical clustering of 2DE gel images (n = 18) revealed a pair of clusters, containing 11 and 7 samples. Previously we used the same specimens to measure biochemical profiles based on cytochrome P450-dependent enzymatic activities and also found that samples were clearly divided into two well-separated groups by cluster analysis. It turned out that groups by enzyme activity almost perfectly match to the groups identified from proteomic data. Of the 271 reproducible spots on our 2DE gels, we selected 15 to distinguish the human liver cytosolic clusters. Using MALDI-TOF peptide mass fingerprinting, we identified 12 proteins for the selected spots, including known cancer-associated species.

**Conclusions/Significance:**

Our results highlight the importance of hierarchical cluster analysis of proteomic data, and showed concordance between results of biochemical and proteomic approaches. Grouping of the human liver samples and/or patients into differing clusters may provide insights into possible molecular mechanism of drug metabolism and creates a rationale for personalized treatment.

## Introduction

Hepatic metastases usually progressively damage liver function and are highly malignant and refractory to conventional therapies [1]. Understanding the molecular and biological mechanisms of colorectal cancer may allow for the development of further therapeutic strategies designed to prevent and treat liver metastases [2], [3]. Furthermore, molecular-based therapies can extend time to liver recurrence after curative resection and may prolong patient survival. The development of more effective and less toxic anticancer strategies also will allow for the personalization of therapeutic regimens according to the molecular features of individual patients [4].

Proteomic studies of liver samples can help to identify specific protein markers for metastases [5]. One of the most frequently used procedures for characterization of the protein components of biological systems is two-dimensional polyacrylamide gel electrophoresis (2DE) in combination with mass spectrometry. Typically, 2DE is used to compare changes in protein level, modification, and degradation between treated and untreated samples [6]. Proteomic changes can be revealed by gel image analysis after visualization by staining and identification of protein species with altered expression or in post-translational states [7].

Most studies based on 2DE analysis of protein profiles in colorectal cancer are conducted on tissue lysate of colorectal adenocarcinoma, adjacent normal colon mucosa, and liver metastases [8]–[10]. Such a strategy is largely limited to abundant proteins (e.g., structural proteins, glycolytic enzymes, annexins, cathepsins, and heat shock proteins) that are overexpressed in several cancers [8]. However, these proteins may hamper the identification of low-abundance proteins, such as membrane proteins, which play a fundamental role in cell signaling, cell-cell interactions, communication, and transport mechanisms, and are drug targets [11]. Targeted approaches associated with the isolation from clinical material subproteomes, such as the secretome, proteasome, plasma-membrane fraction, nuclear matrix, etc., have emerged recently. Subcellular fractionation combined with mass spectrometry techniques is a powerful approach to uncover novel, low abundant, specific colorectal-associated proteins and candidate biomarkers [8]. For example, 1687 protein spots were observed on large format gels (24×33 cm) of a soluble protein fraction of cancerous and normal mucosa tissues and it was shown that the intensity of ≥96% protein spots was scattered within 2-fold differences and >90.5% within 1.5-fold differences [12]. For a human liver cytosolic fraction on 17×24 cm gels, 2-fold fewer protein spots (911 spots) were matched [13]. 2DE image analyses showed that the number of protein spots were significantly changed in primary cancer and hepatic metastatic lesions. Reportedly, the membrane fraction of primary cancer and hepatic metastases demonstrated loss of protein content (i.e., the number of matched protein spots on 2DE gels) compared to the membrane fraction of normal colorectal mucosa [10]. Muto et al. [12] generally reported that the proteome of normal tissue may be more homogeneous than that of tumor tissues.

**Figure 1:**
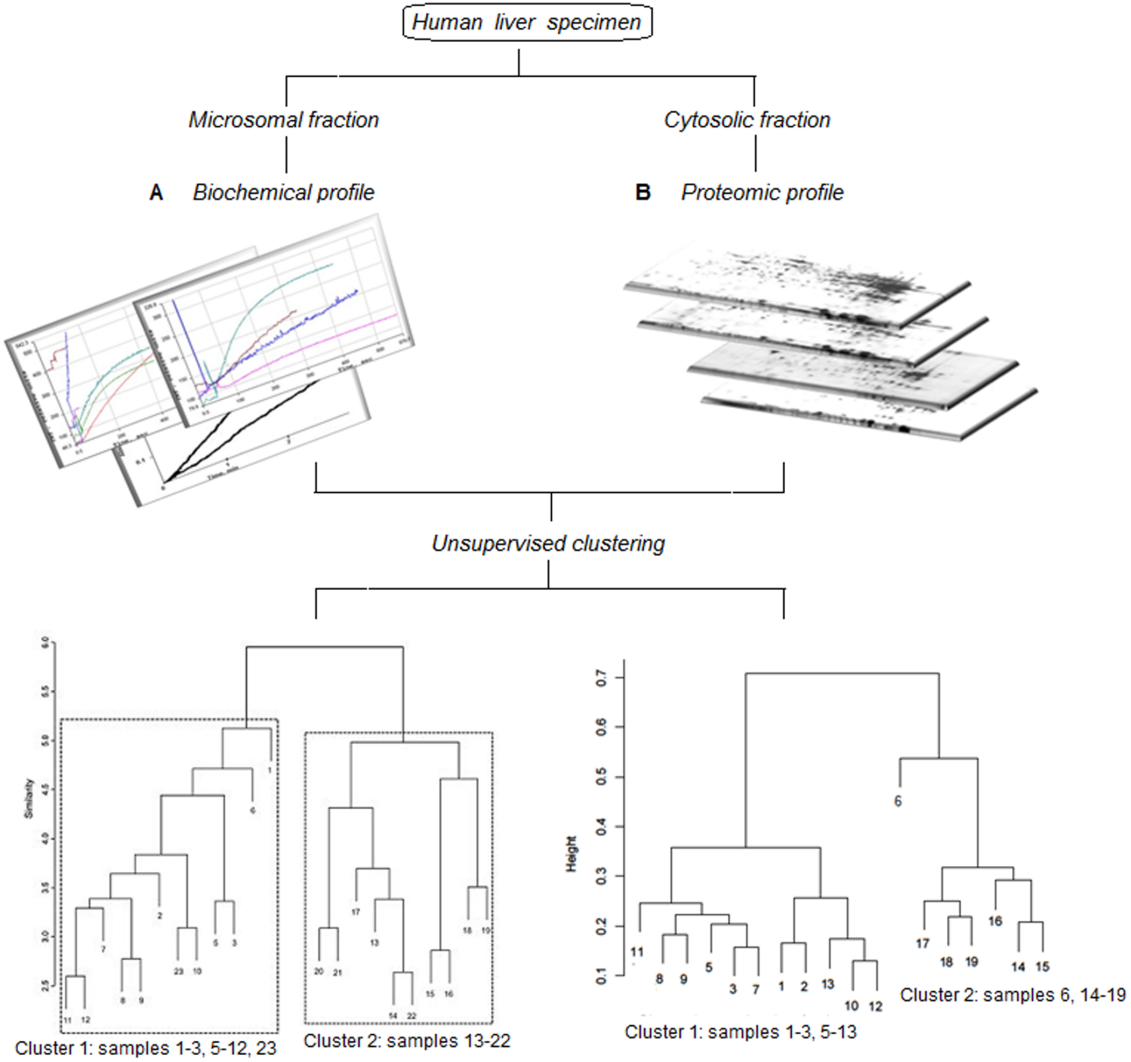
Schematic description of the comparative analysis of human liver biochemical (A) and proteomic (B) profiles. (**A**) The profile included 12 parameters, namely activity of NADPH-cytochrome P450 reductase, cytochrome P450 content and cytochrome P450-dependent monooxygenase activities with marker substrates (Petushkova et al., 2010). (**B**) The profile included 2DE images.

Earlier, we described an approach to discriminate liver microsomal samples into certain classes based on biochemical profiles [14]. In this report, an experimental design to compare the biochemical and proteomic profiles is presented (Figure 1). In the first stage of this approach the biochemical profile was obtained. It included 12 parameters, namely activity of NADPH-cytochrome P450 reductase, cytochrome P450 content and 10 cytochrome P450-dependent monooxygenase activities with marker substrates. Purely from unsupervised statistical analysis of biochemical profiles of human liver microsomes we derived that patterns of the liver monooxygenase system formed two well-separated groups (Figure 1, **A**). It was shown that at least 6 variables were significantly different between two major clusters of human liver microsomal samples having *p*-value <0.05 with Bonferroni correction. In addition, it was found that changes in NADPH-cytochrome P450 reductase activity comprise the main factor, responsible for separation of biochemical profiles between two groups of patients [14].

To correlate the HLC protein profile, as defined by 2DE, with biochemical activities of the microsomal system we used previously collected morphologically normal liver specimens surrounding hepatic metastases of colon cancer. Ultracentrifugation protocol was employed to yield human liver cytosol and microsomal fractions from the same liver specimen. The current study was conducted to compare the sample clusters obtained according to microsomal biochemical activity (Figure 1, **A**) with the respective cytosolic proteomic profiles (Figure 1, **B**). Furthermore, we were interested in the identification of soluble proteins, which may be somehow related to the activity of membrane-bound cytochromes P450.

## Materials and Methods

### Ethical procedures

All samples from residual liver after histological analysis were approved by the Department of Pathological Anatomy of the National Research Center of Surgery, Russian Academy of Medical Sciences (Moscow, Russia). Informed consent was obtained from all patients. Individuals agreed to participate in the study according the local institution’s ethical committee of the National Research Centre of Surgery. Samples have been described in previous publications [14], [15].

### Chemicals

2-[4-(2-hydroxyethyl)piperazin-1-yl]ethanesulfonic acid, phenylmethanesulfonylfluoride (PMSF), 2.5-dihydroxybenzoic acid, ethylenediaminetetraacetic acid (EDTA), nicotinamide adenine dinucleotide phosphate, sodium dithionite, trypsin, and sodium deoxycholate were purchased from Sigma-Aldrich (St. Louis, MO, USA); acetonitrile and trifluoroacetic acid were purchased from ICN Pharmaceuticals Inc. (Costa Mesa, CA, USA); Coomassie Brilliant Blue R-250 was purchased from Fluka (Seelze, Germany). Modified trypsin (catalog no. V511C) was obtained from Promega (Madison, WI, USA). Other chemicals were purchased from Reakhim-Penza, LLC (Penza, Russia).

### 2.3 Tissue samples and preparation

The human soluble liver protein fractions were prepared in our previous study (stored at –80°C prior to usage) from resected and discarded masses of surrounding liver tissues, which were taken from patients (n = 23) undergoing hepatic surgery [14]. Samples of morphologically normal liver (3–10 g,) were obtained from the distal edge of the resection, at least 5 cm off the tumor. Samples were diagnosed by histopathology.

As consisted with conditions of the experiment, we did not take into consideration any personalized information about patients or concerning patient treatment. However, collection of samples was performed according to the following instructions: (1) all patients were under severe cancer disease, which led to the surgery of liver maybe after a prior chemotherapy; (2) no radiotherapy was performed before surgery; (3) no evidence of endocrine or metabolic disease; (4) no severe infection was detected; (4) patients’ dietary requirements were managed by the National Research Center of Surgery to a relatively uniform standard; as a result, exogenous dietary influence on metabolic profiling was limited to the lowest level. The resected samples were placed immediately on ice prior to obtaining the human liver microsomal fraction [14] and HLC. All preparation procedures were performed at 0–4°C. The liver samples were homogenized in two volumes of homogenization buffer, containing 1 mM EDTA, 1 mM dithiotreitol (DTT), 0.1 mM PMSF, and 150 mM KCl using a glass Potter Elvehjem homogenizer with a teflon pestle (Sartorius Stedim Biotech GmbH, Goettingen, Germany). The liver homogenate was successively centrifuged at 10,000×*g* for 20 min and 105,000×*g* for 70 min. The pellet (microsomal fraction) was analyzed as described in our previous study [14]. In the present study, the supernatant was used as the HLC fraction. The protein concentration of the HLC samples was estimated using the Bradford assay [16] with bovine serum albumin as a standard.

### Prefractionation of HLC samples prior to two-dimensional polyacrylamide gel electrophoresis

Aliquot of HLC (200 µL, 13.2±5.6 mg of protein) was mixed with 1 mL of cold 10% trichloroacetic acid (TCA) in acetone (v/v), containing 0.07% mercaptoethanol. After a 3-h incubation at −18°C, the mixture was centrifuged at 20,000×*g* for 10 min (4°C). The supernatant was discarded and the pellet was dissolved in 5 mL of cold acetone, containing 0.07% mercaptoethanol, and centrifuged as described above. The supernatant was discarded and the resulting pellet was used for protein separation by 2DE.

### Two-dimensional polyacrylamide gel electrophoresis (2DE)

2DE of HLC proteins was performed as described by the manufacturer (Bio-Rad, Hercules, CA, USA). For each HLC sample, the resulting pellet was dissolved in 200 µL of rehydration buffer (4 M urea, 2 M thiourea, 4% 3-[(3-cholamidopropyl)-dimethylammonio]-1-propane sulfonate, 50 mM DTT, and 0.5% ampholine). Proteins were loaded by passive rehydration onto 11-cm, nonlinear, immobilized, pH gradient (IPG, pH 3–10) strips overnight (14 h) at 50 V and for a further 30 min at 250 V. Isoelectric focusing (IEF) was performed using the Protean IEF Cell (Bio-Rad) with an applied gradient 250–5500 V for a total of 35,000 V·h. All IEF steps were performed at 20°C. Following IEF, IPG gel strips were equilibrated in equilibration buffer (50 mM Tris-HCl, pH 6.8, 6 M urea, 2% sodium dodecyl sulphate (SDS), 20% glycerol) containing 1% DTT and shaken for 30 min at 50 rpm on an orbital shaker [17]. The IPG strips were then transferred to the equilibration solution, containing 2.5% acrylamide, and shaken for an additional 30 min before separation on a polyacrylamide gel (135×80×1.0 mm, 4% stocking gel, and 12% resolving gel). Separation in the second dimension was performed using the Mini-Protean Dodeca Cell (Bio-Rad) and Tris-glycine buffer (25 mM Tris base and 192 mM glycine), containing 0.1% SDS, at 150 V. Run time was approximately 60 min and upon the exit of bromophenol blue into the buffer, the electrophoretic separation was considered complete.

### Protein visualization and image analysis

Gels were subjected to silver staining [18], gel images were acquired using a GS-800 Calibrated Densitometer (Bio-Rad) and uploaded into the proprietary digital image analysis software GelEditor. It is written in Java and supportsl tools for loading images, automated spot detection based on Laplacian of Gaussian Filter, manual spot detection, matching of protein profiles, and an option to save the reports (Figure S1). The spot intensity on the gel was calculated as the sum of the pixels in a manually detected spot. The GelEditor software can be freely downloaded from www.bioinformatics.ru/geleditor.zip.

### In-gel digestion

The protein spots (∼3 mm^3^) were excised from the gel using modified 250-µL tips and destained with 50 µL of 100 mM K_3_ [Fe(CN)_6_] and 100 mM sodium thiosulfate in a ratio of 1∶1 (v/v) per gel piece at room temperature for 30 min. Afterwards, the gel pieces were washed with water at room temperature and shaken for 15 min at 50 rpm on an orbital shaker. The procedure was repeated three times. Then, the gel pieces were washed twice with 150 µL of 50 mM NH_4_HCO_3_ in 50% acetonitrile at 37°C, shaken for 15 min at 50 rpm on an orbital shaker, and incubated for 15 min in dehydration solution (100% acetonitrile). After the acetonitrile was removed and the gel pieces dried, 8±2.0 µL of trypsin solution (25 ng/µL modified trypsin in 50 mM bicarbonate ammonium) was added and the mixture was incubated at 37°C overnight. Then, 15 µL of 0.7% trifluoroacetic acid were added to each gel piece and the samples were incubated for 2 h at room temperature. The extracted tryptic peptides were used for mass spectrometric analysis.

### Matrix-assisted laser desorption-ionization time-of-flight mass spectrometry (MALDI-TOF MS)

Each mixture of proteolytic peptides (1 µL) was spotted on a MALDI target (600/384 Anchor chip; Bruker Daltonik GmbH, Bremen, Germany) in three replicates and air-dried. For ionization, a solution of 2.5-dihydrobenzoic acid (3 mg/mL) in acetonitrile and 0.7% trifluoroacetic acid (1∶1 v/v) was used. Mass spectra in the *m/z* range of 600–4000 were manually acquired using FlexControl software (Bruker Daltonik GmbH) in the reflection/delayed extraction mode with an accelerating voltage of 25 kV and a 135-ns delay using the Ultraflex II MALDI-TOF MS analyzer (Bruker Daltonik GmbH). All mass spectra represented signals averaging 100 laser shots from one location on a sample spot. From each sample spot, 4–6 mass spectra were acquired. Laser fluency was adjusted above the desorption threshold of the matrix to obtain the best resolution and the highest mass measurement accuracy. Signals with an S/N ratio >6 and a maximum of 100 peaks per spectrum were used to build peak lists with the SNAP algorithm (FlexAnalysis software ver. 2.0; Bruker Daltonik GmbH) and internally calibrated with trypsin autolysis products (*m/z* 842.5094 and 2211.1046 Da, respectively). Resulting peak lists were used to search against the UniProtKB/Swiss-Prot database (UniProt release 2012_09 - September 11, 2012). Identification by peptide mass fingerprinting (PMF) was performed using Mascot software (Matrix Science, Inc., Boston, MA, USA). During the database search, a maximum of one missed cleavage was allowed, a mass tolerance of 80 ppm was used, and variable modifications, such as methionine oxidation and cysteine modification with acrylamide, were taken into account. The appropriated m/z tolerance (80 ppm) was estimated as maximum mass deviation based on statistical distribution of mass errors in all spectra and its standard deviation.

### Statistical analysis

The initial dataset consisted of 19 samples (2DE gels), each characterized on average by 271±99 protein spots/features (Table S1). Gel no. 6 was used as the master gel for the manual spot-to-spot alignment. Since we were interested in comparing the results with prior knowledge on biochemical profiles measured for the same samples [14], we also excluded gel no.4 because in our previous study it was considered as an outlier; so we had a collection of 18 gel images. To ensure study reproducibility and decrease noise sensitivity, we analyzed only qualitative differences between gels in terms of presence/absence of a certain protein spots. Each gel was replicated three times and the best replica was selected by visual inspection of separation quality. Final dataset was presented as a binary matrix **D** consisting of 18 rows (gels images) and 389 columns (spots) where *d_ij_* = 1 if *j*-th spot was present on *i*-th gel, otherwise *d_ij_* = 0. For gel clustering, we used Ward’s method. Distance metric between two binary vectors was defined as number of bits where those vectors differ (also known as Hamming distance). All computations and graphics were performed with R statistical language (www.r-project.org). The source code of the data analysis script can be found in Data Analysis Script S1. To measure the similarity between two data clusterings, we calculated the adjusted Rand index whose value is in the range between −1 and +1, where +1 corresponds to perfect agreement between partitions.

## Results and Discussion

In our experiments all samples were taken from one category of patients: surgically treated for liver metastases arising from colon cancer (Figure 1). Therefore, our aim was not to find differences between norm and cancer, but rather to explore structure inside one-group dataset, describing human liver drug-metabolizing system and cytosol fraction (the present study). In previous study regarding drug metabolism [14] despite relatively modest sample size (n = 22) our results undoubtedly confirmed presence of two clusters in data. We computed silhouette width criteria for cluster validity, which showed that presence of two clusters is confirmed with very high confidence (p<0.0001). Therefore the revealed heterogeneity within one equally treated group of patients was a background for the currently reported proteomic investigation.

### 2DE analysis of HLC

Studies on human liver tissues have been conducted to identify if there are groups of samples distinctive in their proteomes. The HLC proteome was characterized by a representative collection of 19 samples separated by 2DE on middle format gels in three replicates, thus a total of 58 2DE images were examined. The best 2DE replicates were selected from the technical runs according to the separation quality (Figure S2). The selected representative images were converted to the spot lists and further clustered using the unsupervised method. Consistently with our previous work with liver microsomes here we used an unsupervised approach because the clinical information regarding the resected liver samples was unavailable.

**Figure 2:**
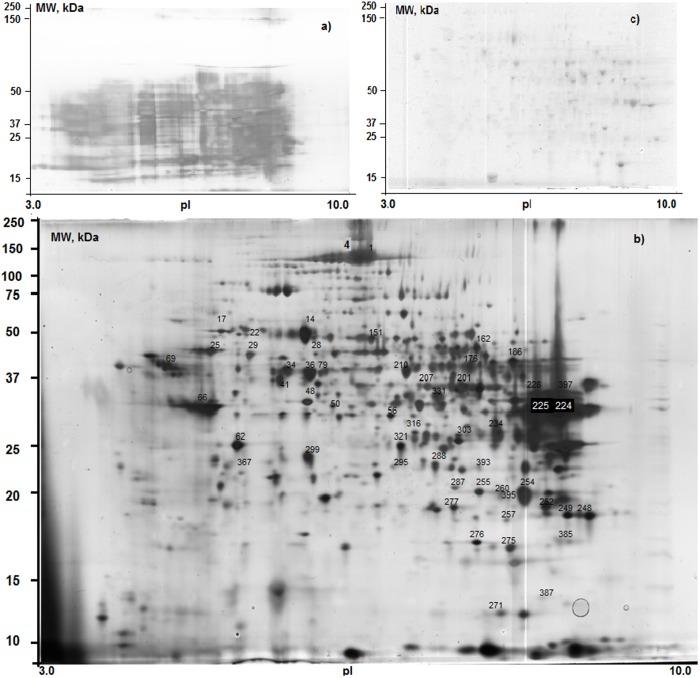
A Typical 2DE images of master gel no. 30 µg of the human soluble liver protein fraction (HLC) was separated by 2DE and visualized by silver staining (***a*** and ***b***) or Coomassie Brilliant Blue staining (***c***): ***a*** **–** HLC before pretreatment with trichloroacetic acid in acetone; ***b*** and ***c –*** HLC after pretreatment. Using Coomassie staining nearly 100 protein spots could be revealed, but using silver staining labeling up to 687 protein spots were automatically detected. Spots nos. 14, 25, 34, 36, 48, 56, 62, 63, 66, 210, 214, 219, 247, 252, 275, 276, 279, 301, 321, 322, and 408 are common for all 2DE gels of 19 human liver cytosol samples.

We rejected some samples due to low quality 2DE separation because of peculiarities in sample preparation (Figure 2). The typical 2DE images of the Ag-stained gel of sample no. 6 before and after prefractionation with TCA in acetone (see Materials and Methods) are shown in Figure 2, ***a*** and ***b***. Without prefractionation, 2DE of cytosol fraction hampers separation of protein spots (Figure 2***a***). Figure 2***b*** is a representative master gel image showing separation of proteins from human liver tissue after TCA/acetone precipitation. This practice is often employed to remove contaminants because it minimizes protein degradation [19], but TCA/acetone precipitation was insufficient to obtain appropriate gel images of the HLC sample nos. 20–23, so we excluded these samples from further analyses. In total, 687 protein spots were resolved on a silver-stained 2DE gel from HLC sample no.6, technical run #1, using spot detection tool of GelEditor software. In addition to the silver staining based on protocol of Shevchenko et al. [18] Coomassie blue staining was also probed in this study and approximately only 100 protein spots were detected on the same gel no.6 (Figure 2***c***).

The obtained gel images were examined to characterize spot reproducibility and variability. We observed that 96% of the protein spots were at the lower boundary of detection limit of silver thiosulfate with a normalized intensity < 0.02 relative units. A medium intensity of 0.02–0.04 units was observed for 2% of spots, while 1% of spots was of high abundance with relative intensity of >0.1 unit.

**Figure 3:**
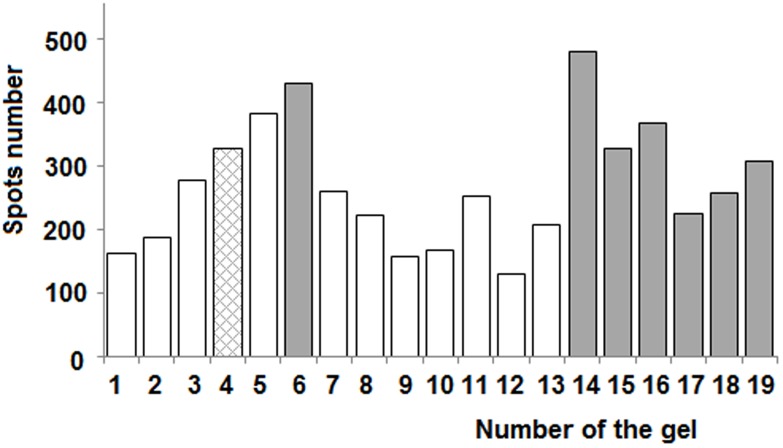
The number of protein spots after manual processing of 19 2DE gel images for the human liver cytosol using the proprietary GelEditor software. Gels belonging to cluster 1 are shown in white columns. Gels belonging to cluster 2 are shown in gray columns. Gel which removed from all subsequent data processing is shown in the shaded column.

Further, we selected the master gel as the best gel with the highest number of spots and minimal range of scattering of the average intensity of each spot. The master gel corresponded to sample no 6. To estimate the technical variation of our experimental setup, we conducted four independent replicative runs of HLC sample no. 6 at different times. At first step of analysis we used number of spots as a rough measure of gel reproducibility. A total of 449, 420, 444, and 406 protein spots were detected in these runs in the ranges of pI 3.5–10.0 and MW 10–250 kDa. The observed average number of spots was 430±20, which was comparable to earlier data obtained from the cytoplasmic fraction of primary human hepatocytes in HepG2 and Hep2B cell lines [20]. The remaining gels contained sufficiently fewer spots, as shown in Figure 3 for 19 HLC samples. In the whole series, the average number of manually detected protein spots was 271±99 (mean ± SD, n = 58). The spot quantities were three-fold lower compared to the earlier reported results of cytosolic fraction analysis of human liver tissue samples [13], [21], [22]. The difference in spot number was probably due to the 2DE setup, as we used 11-cm IPG strips instead of 17 cm, used by Wimmer et al. [13] and Kim et al. [23], and so the gradients for isoelectric focusing were shorter. Kim et al. [23] analyzed tumor and nontumor regions of resected liver cancer tissues by identification of cytosolic proteins using 2DE and MALDI-TOF-MS. They used silver staining for protein detection on the gels and observed up to 1060 spots and identified 127 proteins; they also reported that a problem with the variability of spot intensities was decided using normalized intensities.

To evaluate gel-to-gel variability, we compared the spots between four replicated gel images obtained from sample no. 6 by using the manual spot matching feature of the proprietary GelEditor software (Figure S1). Each replicate was manually aligned to the master image, which contained a maximum number of spots. We found that 102 spots were present in at least three replicates, 80 spots matched one-half of the gels, while 58 spots were present on the master gel only. Finally, 47% of the spots (209 spots) matched all four replicated 2DE images.

**Figure 4:**
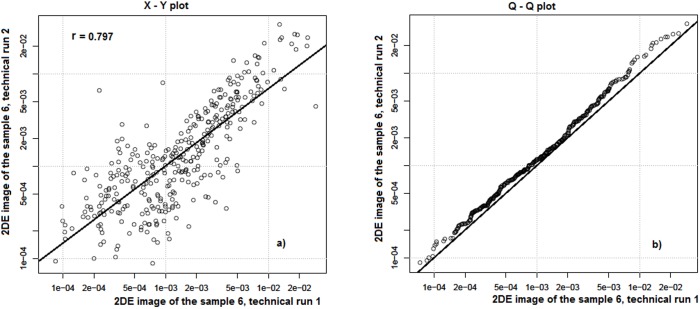
Reproducibility of quantitative data in 2DE images from human liver cytosolic fraction. Normalized spot intensities from two technical runs of the gel no.– Y plot and quantile-quantile (Q – Q) plot. The X – Y plot shows strong correlation between spot intensities with a Pearson correlation coefficient r = 0.797 (**a**). Q – Q plot shows high similarity between spot intensities distribution (**b**). Intensity of each matched spot on the gel was normalized per overall intensity of matched spots within this gel.

To determine the reproducibility of 2DE, we investigated technical runs of the HLC sample 6, analyzed the normalized intensities of each spot, and plotted those against each other on an X–Y and a quantile - quantile (Q–Q) plots [24]. The X–Y plots for the sample no.6, technical run #1 (master gel) compared with the same sample, technical run #2 show a Pearson correlation coefficient r = 0.797 (Figure 4***a***). We performed such analysis for all four technical runs of the gel 6 and determined the average Pearson’s correlation coefficient which was equal 0.72±0.06 (mean±95% confidence interval). The magnitude of the correlation coefficient r>0.7 suggests a strong correlation, while Q-Q plots were used as a graphical tool for comparing two distributions to each other. Two data sets would be considered identical if the points in the Q-Q plots lie close to a regression line *y = x*. As shown in Figure 4b this criterion is perfectly fulfilled for HLC proteins.

**Figure 5:**
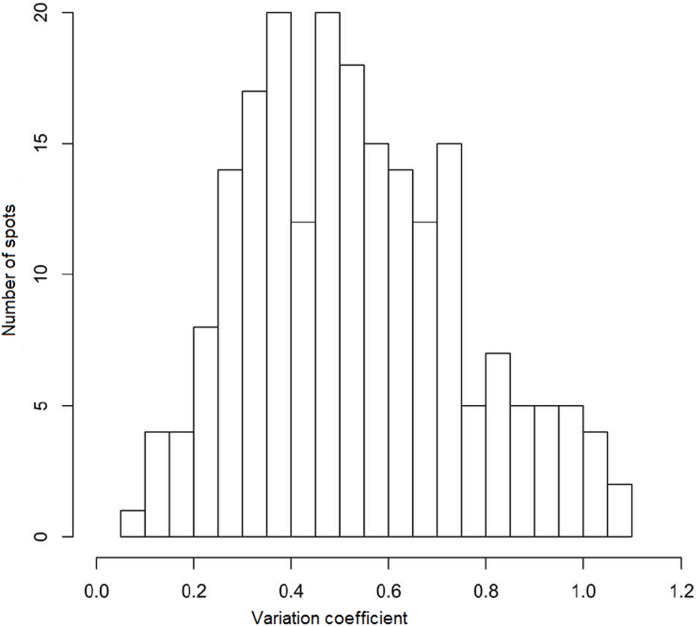
Coefficient of variation for 207 matched spots on the master-gel after manual matching of four 2DE gels using the proprietary GelEditor software for the human liver cytosol (sample 6). Intensity of each matched spot on the gel was normalized per overall intensity of matched spots within this gel.

The highly reproducible spots (209) were used to estimate the spot intensity variation across four replicates for HLC sample no 6. In the coefficient of variation (CV) distribution histogram (see Figure 5), 65% of spots were characterized by a CV <0.6. This degree of variation between the replicated gels was comparable to the CV observed between normal tissue vs. various cancer stages [9], [25]. For example, Shi et al. [9] observed an average CV of 62–69% in 1223 spot features from 18 2D-DIGE spot profiles amongst different sample groups (normal colon mucosa, primary colorectal cancer, and liver metastases). The authors found that both primary and secondary tumors displayed a significantly higher degree of spot volume variations than the normal colon.

Generally, the CV depends on the type of biological material, as well as sample preparation and spot detection approaches [26], [27]. In silver staining, the matching spots prepared and run under identical conditions vary in intensities; therefore, it is difficult to achieve reproducibility even with parallel replicates [26]. Therefore, as our gels were poorly reproducible in the intensities of the matching spots, we did not use intensity values for further analysis, but converted the spots into binary format: either there is a spot, or not.

### 3.2 Unsupervised clustering of the gels and microsomal samples

**Figure 6:**
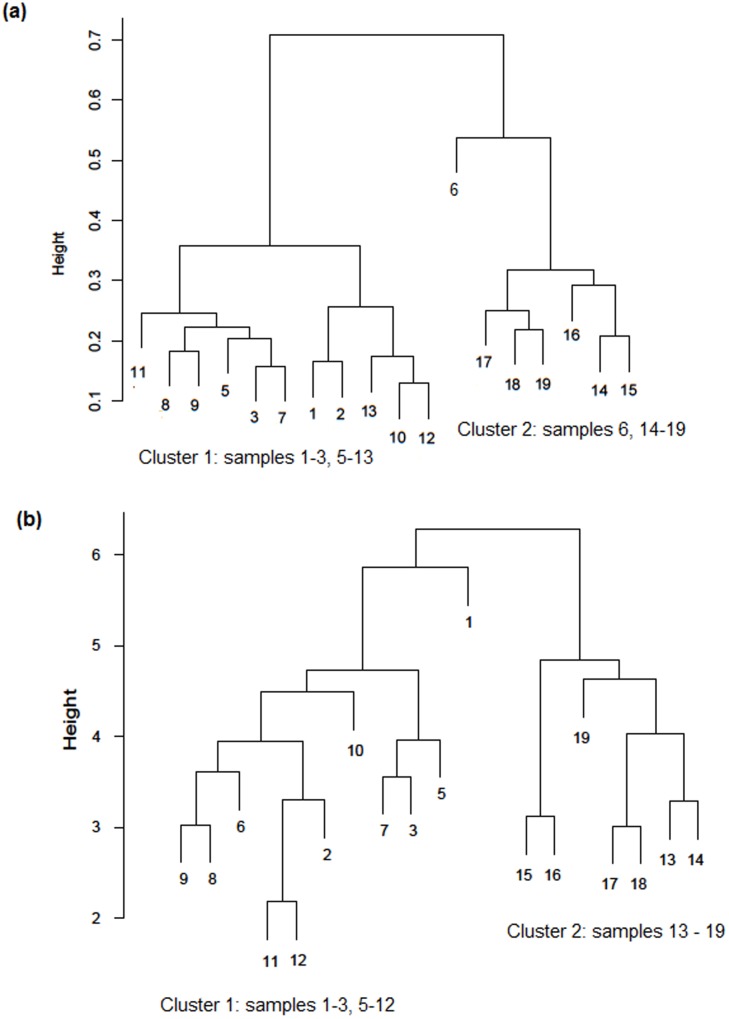
Hierarchical clustering applied to 2DE gel images of the human liver cytosol (a) and cytochrome P450 enzymes activities (mol/min/nmol P450) of the human liver microsomes (b). Two major clusters can be seen.

We used cluster analysis to separate gels and elucidate groups in our collection of liver samples. Initially, spots on each 2DE gel were collated to the spots on the master gel (no. 6#1) using Perl script (Data Analysis Script S2). Our results were formatted into a table in which the rows indicated the protein spots and the columns represented HLC samples. If the protein spot was present on the HLC gel and master gel, the cell value was set at “1”, otherwise if the spot was absent on the next HLC gel, it was set at “0”. We performed hierarchical cluster analysis of the data matrix using Ward’s method coupled with the Hamming distance metric. Clustering of 2DE gels of the HLC samples was visualized as a dendrogram (Figure 6***a***). Two major clusters were clearly distinguished: the first (cluster no. 1) was formed by gels nos. 1–3, 5, and 7–13, while the second (cluster no. 2) included gels nos. 6 technical run #3, and 14–19. So, Ward’s method yielded two groups of liver samples. Interestingly, the histogram in Figure 3 preliminarily pointed to the existence of two clusters for the HLC samples. The average number of manually detected protein spots on gels from clusters nos. 1 and 2 were 219±72 and 342±91 (mean ± SD), respectively; however, the difference in the number of spots was statistically insignificant (*p>*0.05, n = 18).

According to our previous data, human liver microsomes also segregated into two groups [14]: the first contained samples nos. 1–3, 5–12, and 23, whereas the second contained samples nos. 13–22 (Figure 1**A**). Thus, there was a correlation between sample clustering emerged from the two absolutely different experimental approaches.

The Rand index was 0.58, indicating a significant match [27] between biochemical and proteomic data. A slight difference between group composition of the HLC samples and the human liver microsomal (drug-metabolizing) system was found. These changes were particular to HLC samples nos. 6 and 13, assigned to cluster nos. 2 and 1, contrary to their previous clustering by biochemical profiling. As can be seen from the dendrogram in Figure 6***a***, gel no. 6 (technical run 3 of the master gel) can be formally attributed to cluster no. 2, as it is located at a sufficient distance from the centroid of this cluster.

Due to problems with 2DE, the present study included 18 HLC samples vs. 22 microsomal fractions in our previous research [14]. We re-used biochemical data for hierarchical clustering with only 18 (instead of 22) corresponding microsomal samples and obtained consistent results. Again, two groups were observed: sample nos. 1–3 and 5–12 in cluster no. 1, and sample nos. 13–19 in cluster no. 2 (Figure 6***b***). As seen in the dendrograms (Figures 6 ***a*** and ***b***), the human liver samples and/or patients fell into the same subgroup within the same cluster. For example, samples nos. 3 and 7 were occupied neighboring leaf of cluster no. 1 both in biochemical and proteomic analyses, while samples nos. 8 and 9 were neighboring within cluster no. 1, and samples nos. 17 and 18– within cluster no. 2.

We attempted to elucidate the characteristic features for the clusters. The number of protein spots was not significantly different, so the number of the different protein species coincided in both groups. These results indicated, that even under changing biochemical conditions, the number of proteins is generally preserved in the liver cytosol.

Next, we analyzed changes in the intensity level of the spots, which contributed to the differentiations between clusters. Differences between the two HLC groups shown in Figure 6 ***a,*** were observed in 15 protein spots. In details, it appeared that in cluster no. 1, there were three spots (## 1, 4, and 8) that were absent in cluster no. 2.

Although there were no significant differences in the number of spots between clusters, it was observed that gels from cluster no. 1 generally contain less number of spots than gels from cluster no. 2. It was apparent already from the Figure 3, that clusters separate themselves in accordance with the number of spots. In a biochemical survey, we observed the higher activity of drug metabolizing enzymes for the samples of cluster no. 1, where the number of spots was lesser. Vice-a-versa for the gels with larger number of spots in cytosolic fraction the activity of microsomal enzymes diminished. It could be that there is no functional isolation between cytosolic and microsomal fraction. The deficiency of microsomal activity could be compensated by increased activity and/or content of cytosolic proteins.

We identified the particular forms of the proteins (2DE spots), which contributed to the differentiation between clusters of HLC samples. As mentioned already the differences between clusters shown in Figure 6***a*** and/or differences between two groups of HLC were observed in fifteen 2DE spots, three of which were specific for cluster no. 1 (## 1, 4 and 8 in Figure 2***c***). The rest twelve spots were present only in cluster no. 2 but not in cluster no. 1 (## 28, 50, 248, 253, 257, 260, 295, 367, 379, 381, 378 and 395).

The clustered gels were relatively similar in patterns of the differentially presented spots. Of 219 spots observed on the 2DE gel images from cluster no. 1, 13% of the spots were present on each gel, while those relating to cluster no. 2, practically the same portion (16%) of the spots were reproducible in all of the images in the group. From this observation we expect that the observed differences between clusters were not the consequence of individual variability between patients, but rather due to the variability between the cohorts specifically responding to chemotherapy exposure. On the other hand, the stratification of the cohorts can be explained by the different susceptibility to the chemotherapy or some patients may have lacked presurgical treatment.

### Protein identification by MALDI-TOF mass spectrometry

Spots indicating differences between clusters were excised from some gels and identified by MALDI-TOF-MS. For each protein spot, we acquired 6–8 MALDI-TOF mass spectra from each of the two replicated positions on the MALDI target per protein spot. By averaging the *m/z* values in spectra the greatest MASCOT score for protein matching and the highest percentage of sequence coverage were selected (Figure S3). All of these spots were also analyzed using Mr and pI (Table I). Approximately 90% of the identified proteins had sequence coverage exceeding 25% (47±18%). In 44% of the cases, the identified proteins were the single candidate with significant score (173±85) leading to their unambiguous identification. Among 16 proteins presented in Table 1 four proteins, namely pseudopodium-enriched atypical kinase 1 (Q9H792, spot ID # 4), quinone oxidoreductase (P04406, #248), glyoxylate reductase/hydroxypyruvate reductase (Q9UBQ7, #253), and carbonic anhydrase II (P00918, #260) exhibited a score lower of 70. These identifications were confirmed as these proteins migrated within a range consistent with expected mass and pI values. Note, that three of these proteins were secondary residents in the spot identified at the background of the major protein species. For example, spot #253 contained two proteins: fructose-bisphosphate aldolase B (P05062) and glyoxylate reductase/hydroxypyruvate reductase (Q9UBQ7), which were identified with the score 171 and 59, respectively.

**Table 1:** Proteins differentially presented in cluster 1 vs cluster 2 of the human liver cytosol.

Spot ID	Protein name	Assession number^a^	Gene name	MW, Da	pI	Mascot score^b^	Seq.cov, %	Molecular class
1	*Carbamoyl-phosphate synthetase I	P31327	CPS1	164835	6.3	338	34	Enzyme: Ligase
4	*Pseudopodium-enriched atypical kinase 1	Q9H792	PEAK1	192986	6.46	60	15	Enzyme: Transferase
28	**Heat shock 70 kDa protein 1A/1B	P08107	HSPA1A	70009	5.48	118	35	Chaperone protein
28	**Bifunctional epoxide hydrolase 2	P34913	EPHX2	62575	5.91	80	23	Enzyme: Hydrolase
50	**Selenium binding protein 1	Q13228	SELENBP1	52358	5.93	261	61	Protein transport
248	**Glyceraldehyde-3-phosphate dehydrogenase	P04406	GAPDH	36030	8.57	103	59	Enzyme: Oxidoreductase
248	**Quinone oxidoreductase	Q08257	CRYZ	35185	8.56	68	49	Enzyme: Oxidoreductase
253	**Fructose-bisphosphate aldolase B	P05062	ALDOB	39448	8.01	171	59	Enzyme: Lyase
253	**Glyoxylate reductase/hydroxypyruvate reductase	Q9UBQ7	GRHPR	35646	7.01	59	42	Enzyme: Oxidoreductase
257	**Superoxide dismutase [Mn], mitochonderial	P04179	SOD2	24707	8.35	140	53	Enzyme: Oxidoreductase
260	**Carbonic anhydrase II	P00918	CA2	29228	6.87	64	30	Enzyme: Lyase
260	**Carbonic anhydrase I	P00915	CA1	28852	6.59	140	57	Enzyme: Lyase
295	**Phenazine biosynthesis-like domain-containing protein	P30039	PBLD	31765	6.06	256	81	Enzyme: Isomerase
367	**Nicotinamide N-methyltransferase	P40261	NNMT	29555	5.56	105	39	Enzyme:Methyltransferase
387	**L-xylulose reductase	Q7Z4W1	DCXR	25897	8.33	90	43	Enzyme: Oxidoreductase
395	**Triosephosphate isomerase isoform2	P60174	TPI1	30772	5.65	265	79	Enzyme: Isomerase

a Protein accession number as SwissProt at http://www.expasy.org/uniprot.

b MASCOT score from MASCOT protein database search at http://www.matrixscience.com, where the score >56 is statistically significant (p<0.05).

^*^The proteins that had been identified in the gels of cluster 1 and are absent in gels of cluster 2.

^**^The proteins that had been identified in the gels of cluster 2 and are absent in gels of cluster 1.

Table 1 presents the results of the identification performed by peptide mass fingerprinting (PMF). PMF spectra should be used cautiously, as identification results vary with tolerance value [28], [29]; and peptide mass tolerance should receive a special attention regarding such parameters of PMF search engines as taxonomy, cleavage enzyme, the number of peptides matched, etc. Earlier we observed that human liver microsomal protein identification results show the dependency with a maximum at 0.15 Da (120 ppm) [15], [30]. Herein applying the same method for HLC protein identification we obtained that peptide mass tolerance value achieved optimum at 60–90 ppm.

Among the proteins specific to the samples of cluster no. 1 carbamoyl phosphate synthetase I was normally found in hepatocytes. Cardona et al. [26] have shown that the development of small-intestinal adenocarcinoma is associated with colorectal cancer and loss of this protein may be of use to diagnose difficult cases. In the HLC samples belonging to group 2, following proteins were identified: carbonic anhydrase I (CA1, P00915), dicarbonyl/L-xylulose reductase (DCXR, Q7Z4W1), and selenium-binding protein 1 (SBP1, Q13228). CA isoforms have an important role in cancer development and altered CA1 protein levels can support the protective effects of changes in diet and vegetable consumption against colorectal cancer [31]. CA1 is down-regulated in cancerous vs. normal tissues and can be used as candidate prognostic biomarker to indicate good survival prediction for colorectal cancer patients [32]. DCXR has been proposed as a potential biomarker of human adenocarcinoma [33]. Differentially expressed SBP1 also demonstrated anti-cancer potential as its overexpression in HCT116 cells induced H_2_O_2_-mediated apoptosis, inhibited cell migration in vitro, and inhibited tumor growth in nude mice [34]. SBP1 suppression may contribute to the rapid progression of colorectal carcinoma, while higher SBP1 level is associated with differentiation of normal colonic epithelia and may be a positive prognostic factor for survival in stage III colorectal carcinoma [35], [36].

2DE gels of all 18 HLC samples shared 21 common protein spots (see legend for Figure 2***b***), while identification by MALDI-TOF PMF was acquired only for 13 spots of sufficient intensity. Among them there were: malate dehydrogenase cytoplasmic (P40925), aldehyde dehydrogenase 1 (ALDH1, P00352), heat shock cognate (P11142), biliverdin reductase B (P30043), and aminoacylase-1 (Q03154). The amount of proteins listed above was higher in colorectal cancer cells vs. normal colorectal epithelial cells and may present a clinically useful prognostic biomarker of colorectal cancer [37]–[39]. For most of the detected spots no significant difference was observed between normalized intensities on 2DE gels belonging to clusters nos. 1 and 2. For example, the intensities of spot #48 (aminoacylase-1) were 0.0076±0.037 and 0.0072±0.0021, in clusters nos. 1 and 2, respectively. The same situation was also observed for spot #275 (biliverdin reductase B) –0.0128±0.0149 (cluster no. 1) and 0.0098±0.0040 (cluster no. 2). Nevertheless, the change in the staining intensity of one particular spot deserved attention: the intensity of spot #210 (ALDH1) was 0.0174±0.0051 in cluster no. 1 and 0.0114±0.0051 in cluster no. 2. The difference in spot intensities was statistically significant (*p*<0.001, n = 18) and indicated that ALDH1 was 1.5-fold over-expressed in HLC samples of cluster no. 1 compared with its level in HLC samples of cluster no. 2. Lohberger et al. [40] demonstrated that cancer cells with high ALDH1 level were more resistant to commonly used chemotherapeutic agents, such as doxorubicin, epirubicin, and cisplatin, than cells with low ALDH1 amount.

The obtained data showed that the division of liver cytosolic fractions into two groups correlated with the segregation of liver microsomal fractions obtained earlier from the same samples. Characterization of the changes specific to the biochemical and proteomic profiles of human liver samples from patients with the same diagnostic category (i.e., colorectal cancer liver metastases, which may have led to hepatic surgery after prior chemotherapy) may suggest poor survival prediction in cluster no. 1 patients because of two reasons. First, human liver microsomes and/or the patients belonging to this cluster were characterized by a significant increase in activity of phase I metabolizing enzymes vs. activities of drug-metabolizing enzymes in microsomes from cluster no. 2 [14]. It was shown that higher enzymatic activities lead to lower treatment efficacy influenced (decreased) the survival time after chemotherapy [41], [42]. Second, in HLC samples from group 1, the enzymes CA1, SBP1, and DCXR were absent. Down-regulation of these proteins serves as prognostic biomarkers for poor survival prediction in colorectal cancer patients [33], [35], [36].

### Concluding remarks

This functional proteomic study integrated multi-parametric data from two sets of experiments. First, we performed biochemical analysis of liver microsomes, while another set was produced by 2DE analysis of cytosolic fractions. We observed that both sets of experiments were concordant, as samples were clustered in similar ways. Notably, the unsupervised method was used to unravel the clusters; therefore, results of the biochemical experiments did not affected sample segregation in proteome profiling.

Earlier, we observed significant changes in biochemical activities and explained them as different susceptibilities and/or expose patients to pre-operative chemotherapy. In the current follow-up study, the proteomic analysis confirmed two classes of samples and enabled the identification of proteins that were altered concordantly with changes in activity of phase I drug-metabolizing enzymes.

We introduced the application of unsupervised learning to compare the biochemical properties of hepatocytes and changes of the amount of particular forms of the proteins at the proteome level. The presented biochemical-to-proteome profiling approach is essentially different from the conventional proteomic studies, in which samples are attributed to the classes determined by clinical diagnoses.

## Supporting Information

Figure S1 GelEditor software: Brief Description and application.
(PDF)

Figure S2 2DE images of human liver cytosolic fraction (19 samples). 30 µg of the human soluble liver protein fraction after pretreatment with trichloroacetic acid in acetone separated by 2D-PAGE were visualized by silver staining.
(PDF)

Figure S3 Mass spectrometric characterization of proteins differentially presented in cluster 1 vs cluster 2 of the human liver cytosol: a) Fructose-bisphosphate aldolase B; b) Carbamoyl-phosphate synthetase I; c) Glyceraldehyde-3-phosphate dehydrogenase; d) Glyoxylate reductase/hydroxypyruvate reductase; e) Selenium binding protein 1; and f) Superoxide dismutase [Mn], mitochondrial. (A) MALDI-TOF mass spectra of the tryptic digests of the spots on 2DE gels of human liver cytosolic fraction. Labeled peaks (*) correspond to the matched peptides of identified proteins. (B) The sequence coverage of identified protein. Matched peptides shown in bold red.
(PDF)

Table S1 Normalized intensities of the spots on 2DE gels of human liver cytosolic fraction.
(XLS)

Data Analysis Script S1 R script for statistical data analysis (process.r).
(R) Click here for additional data file.

Data Analysis Script S2 Perl script for spot preprocessing (match_gels.pl).
(Pl) Click here for additional data file.
