# Supplementary material for: Applying of Hierarchical Clustering to Analysis of Protein Patterns in the Human Cancer-Associated Liver
Source: PLoS One. 2014 Aug 1;9(8):e103950. doi: 10.1371/journal.pone.0103950 (PMC4118999; doi:10.1371/journal.pone.0103950)
Supplement: Figure S1 — GelEditor software: Brief Description and application. (PDF) [file pone.0103950.s001.pdf]

Figure S1. GelEditor software: Brief Description and application

The input data format of the software requires a set of 2DE gel images (TIFF or PNG) for the protein signal to be studied. The user is presented with the data in a graphical user interface, which shows a window for the signal and gel images, a result window, and a table containing the external variables.

2DE gel images analysis begins using automated detection of staining spots and their shape. Staining area of the spot is installed by the definition of the distinction between "dark" and "light" the pixels by using special tools. The user can interactively define a region of interest and adjust detection of the most abundant protein spots using options “Spot width” and “Sharpness spot border”. Options "Volume" and “Min Height/Width ratio” set the minimum size of the spots and the spots with the area below this value will not be identified.

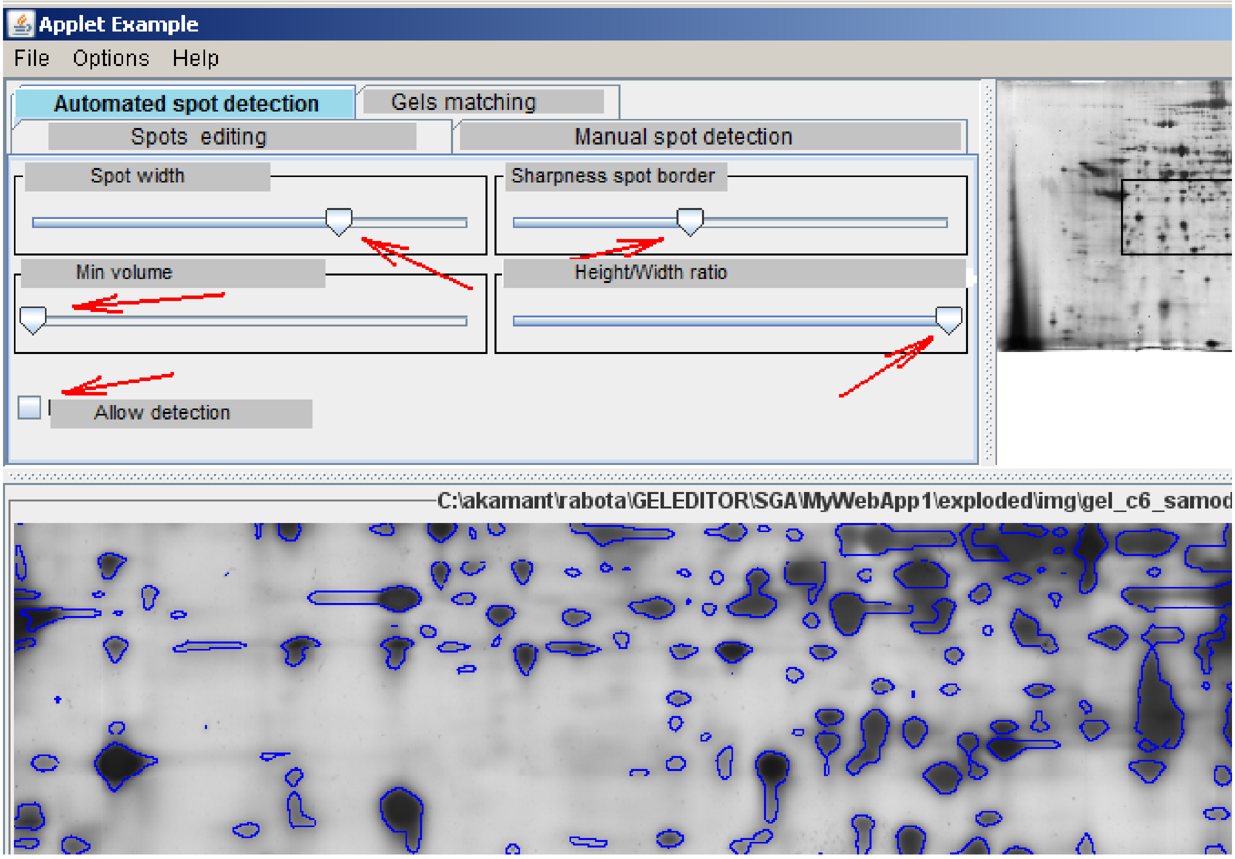

Mode “Manual detection” enables spot editing, and creates a report where all detected spots are automatically assigned a unique number. Report contains information about the spot intensity as the sum of the pixels in a particular spot and volume (%).

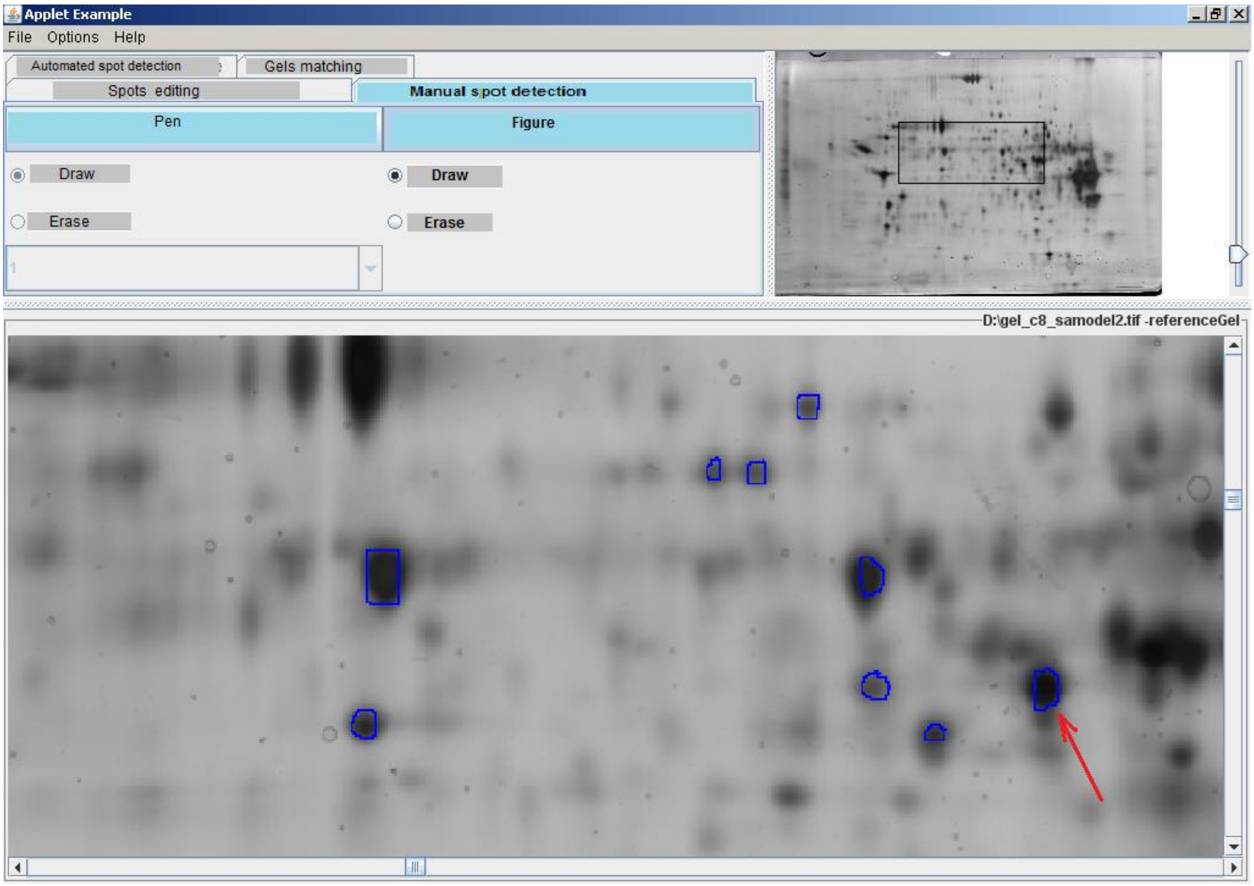

When the user saves the results of manual spot detection the contours of the spots become ovals.

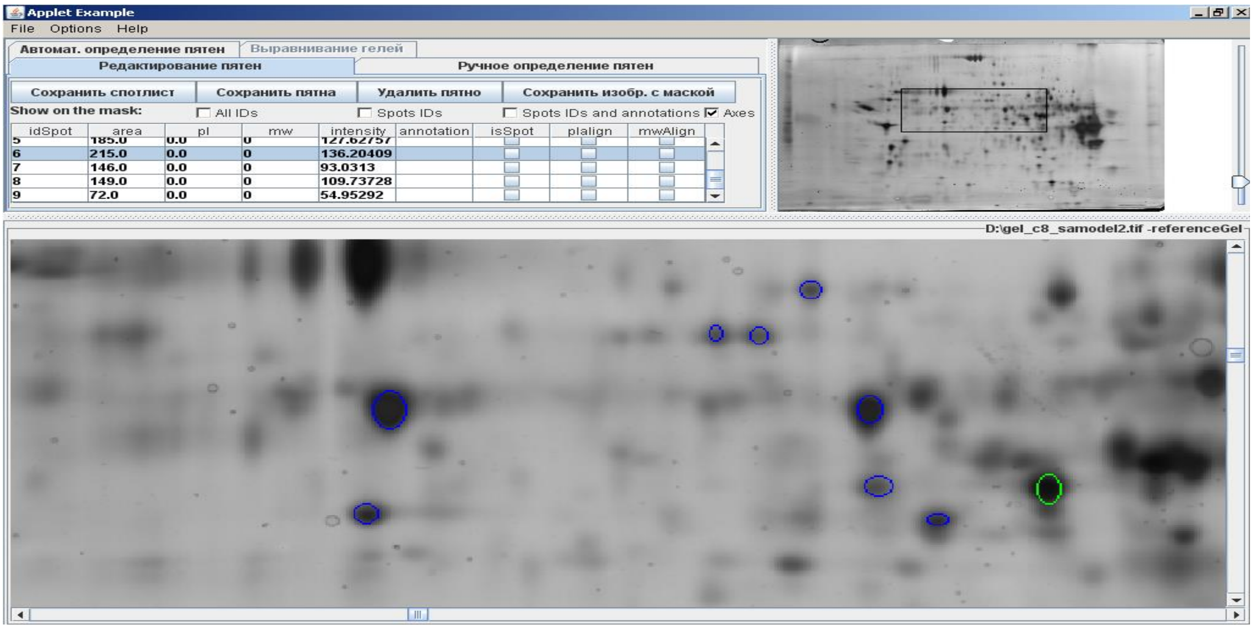

The user can edit the contours of the spot of interest (green stroke appears around this spot) using keyboard keys: **Home** (increase vertical); **End** (decrease vertical); **Ins** (zoom in horizontal), and **Del** (zoom out horizontal).

Alignment of the 2DE gel images is done by manually aligning all images to a reference image (master gel).

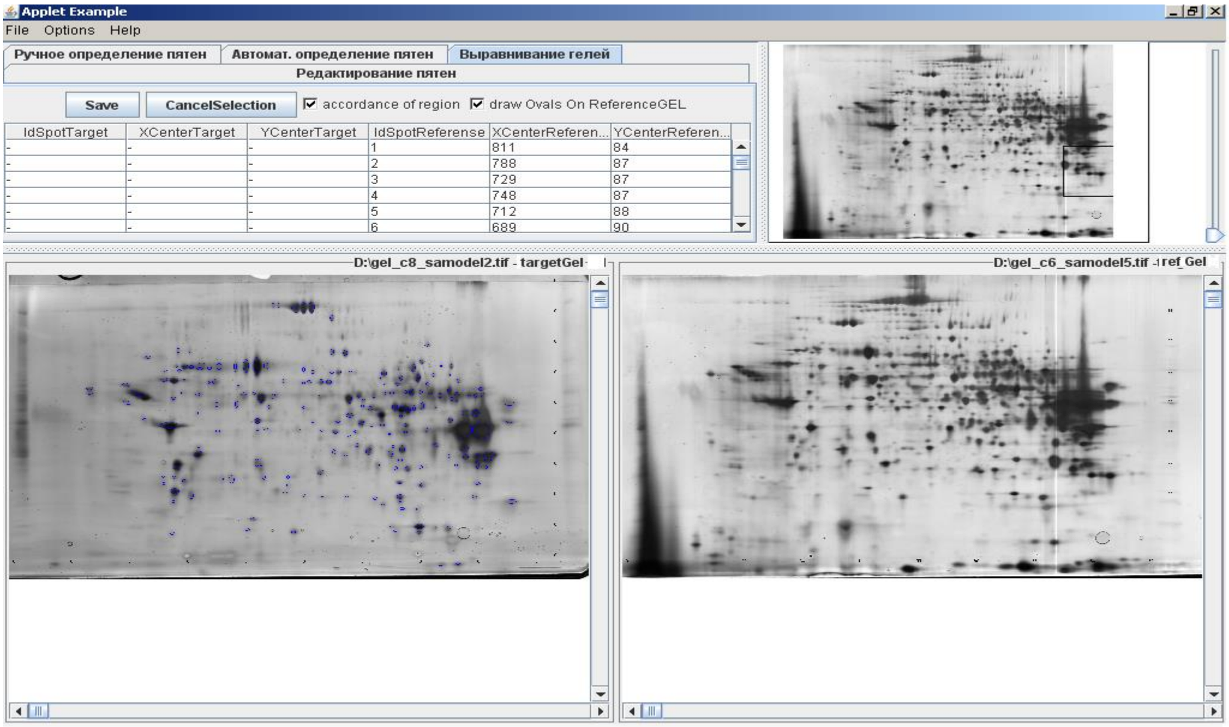

Spots which are characterized by the same X – Y values have one color (e.g., blue) on reference and target 2DE gel images.

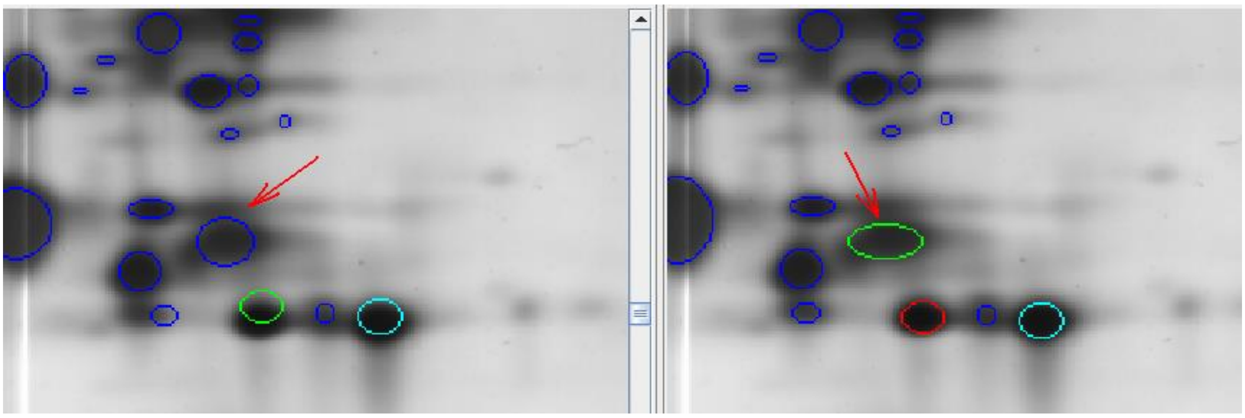

The results of the spot detection and matching are saved as the text files and can be loaded again by the GelEditor software or imported into Excel worksheet for further postprocessing.
