# Supplementary material for: Applying of Hierarchical Clustering to Analysis of Protein Patterns in the Human Cancer-Associated Liver
Source: PLoS One. 2014 Aug 1;9(8):e103950. doi: 10.1371/journal.pone.0103950 (PMC4118999; doi:10.1371/journal.pone.0103950)

Appendix 2. 2DE images of human liver cytosolic fraction (19 samples). 30 μg of the human soluble liver protein fraction after pretreatment with trichloroacetic acid in acetone separated by 2DE were visualized by silver staining.

Sample 1

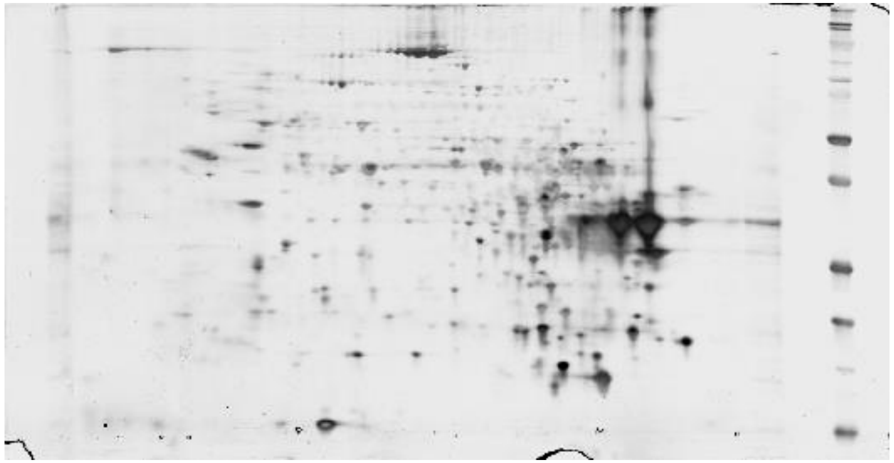

Sample 2

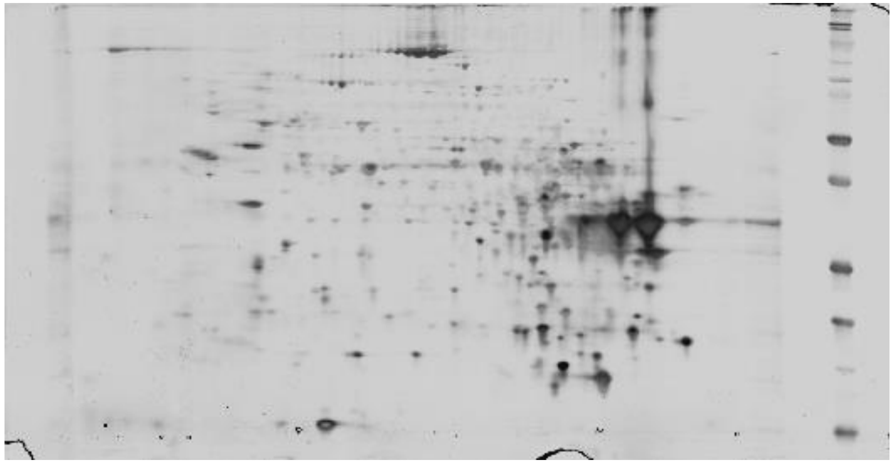

Sample 3

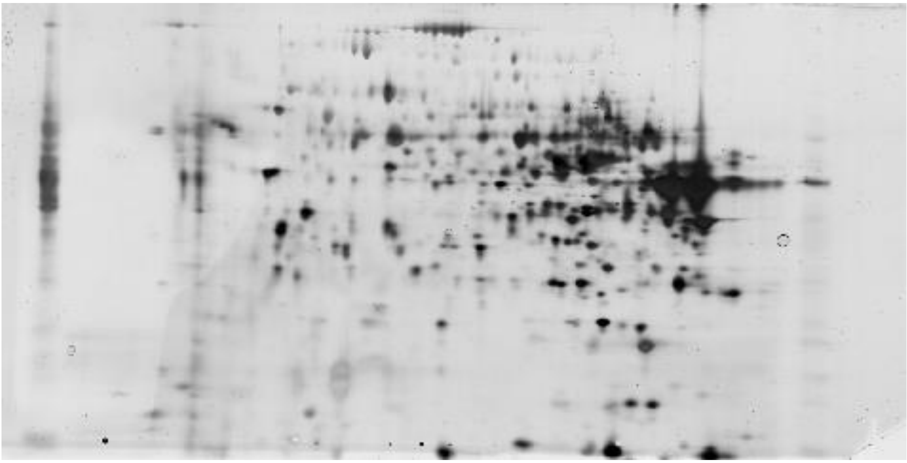

Sample 4

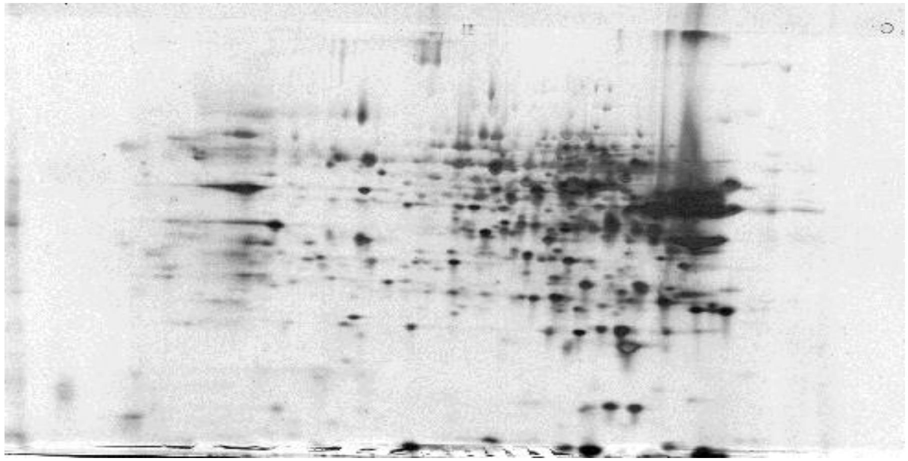

Sample 5

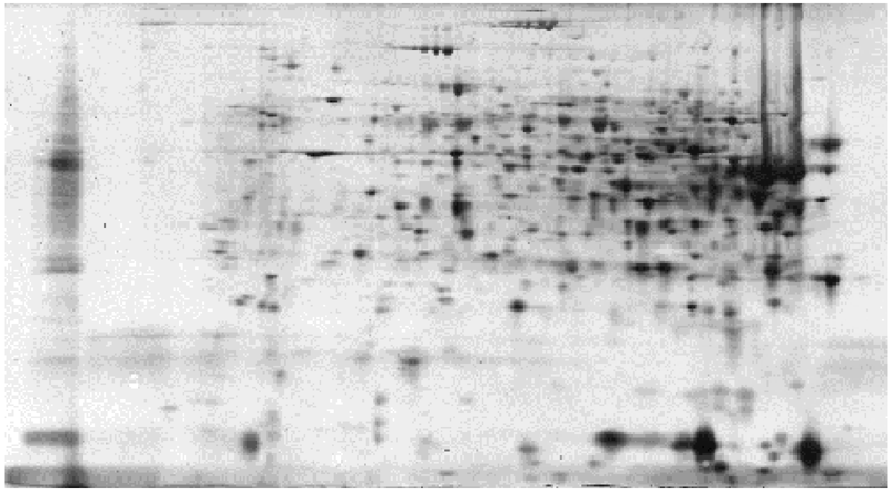

Sample 7

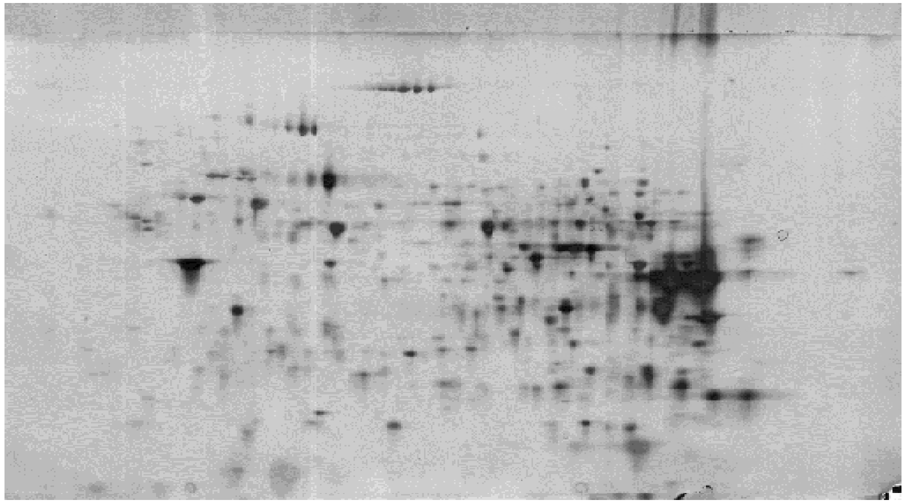

Sample 6 (technical run 2)

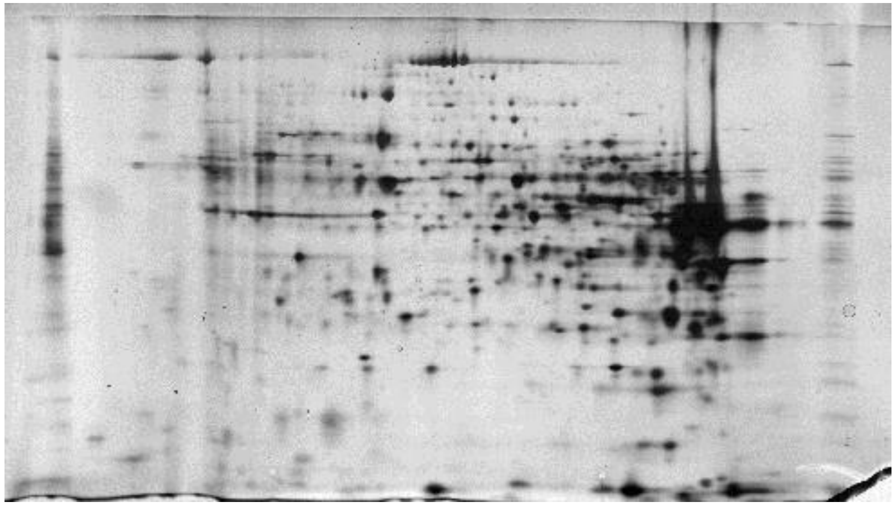

Sample 8

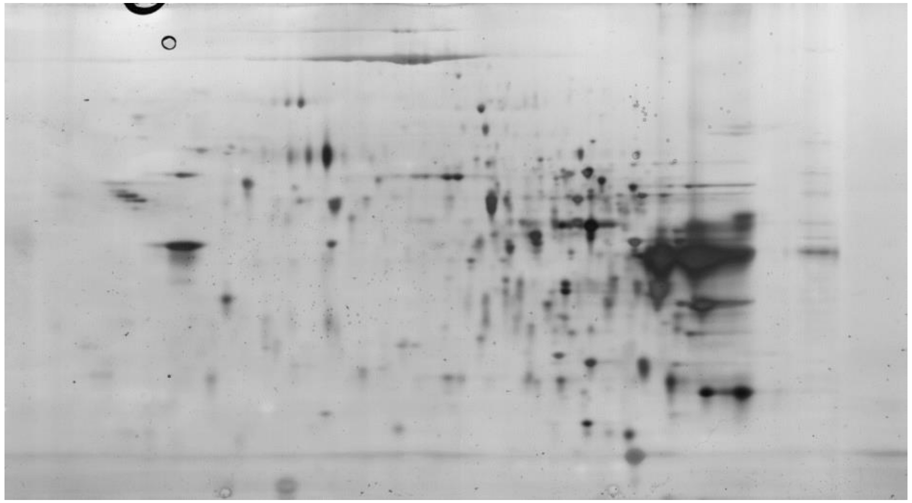

Sample 9

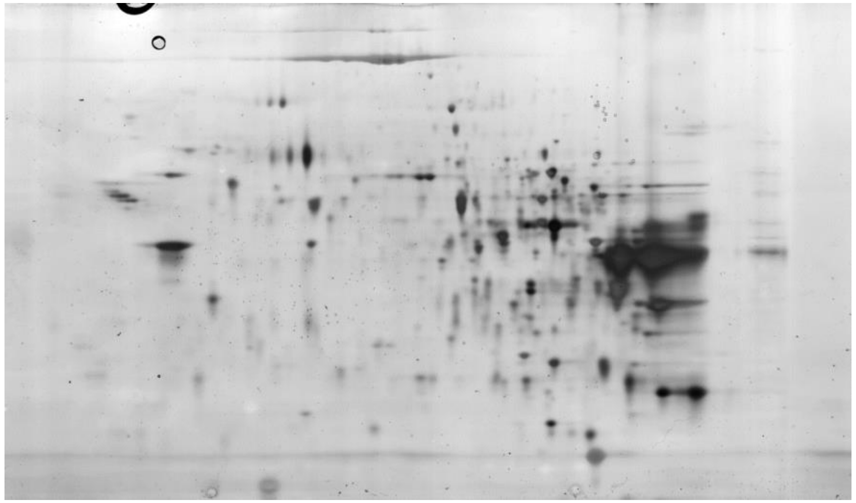

Sample 11

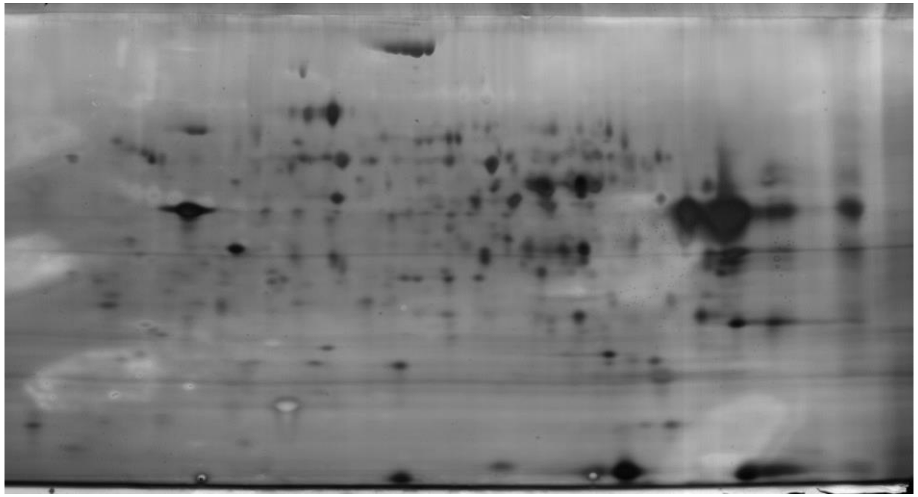

Sample 10

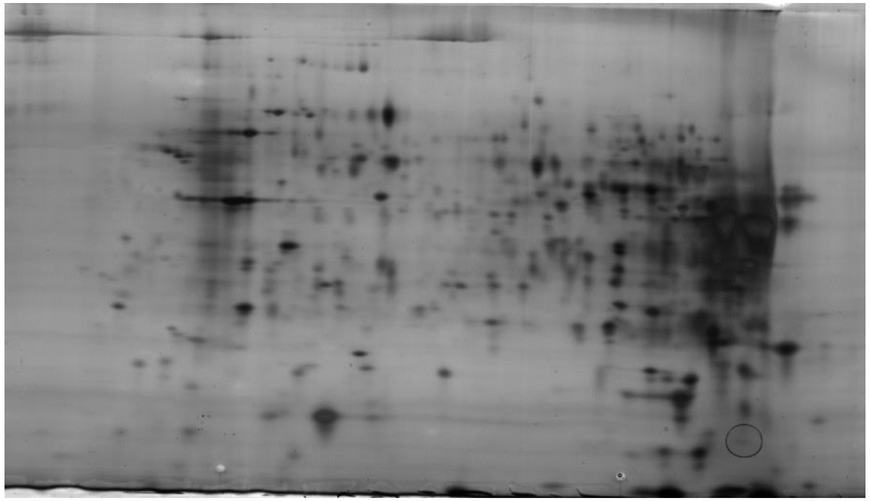

Sample 12

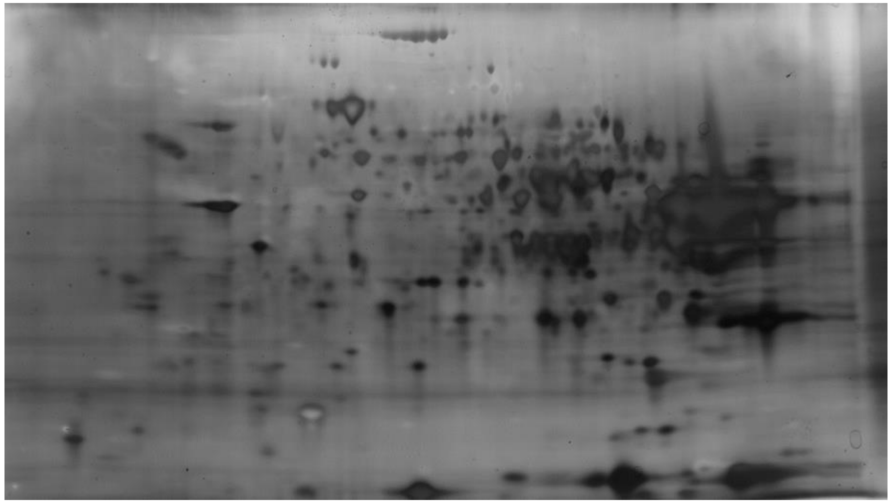

Sample 13

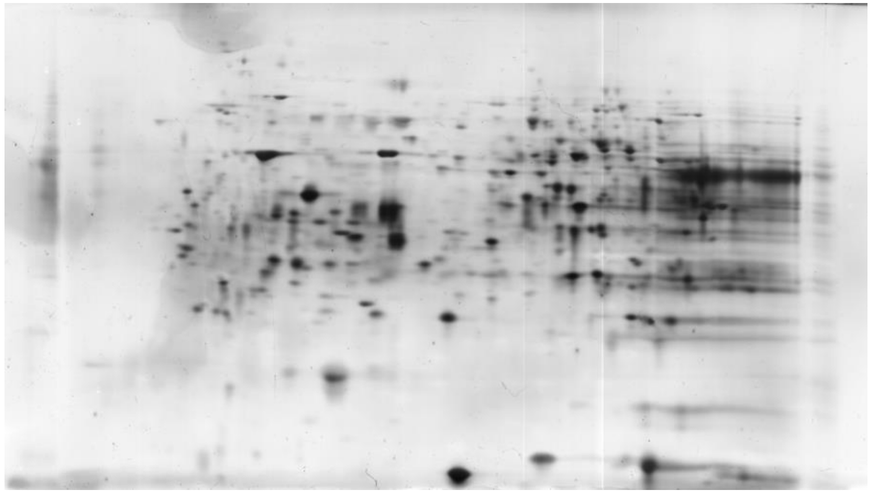

Sample 15

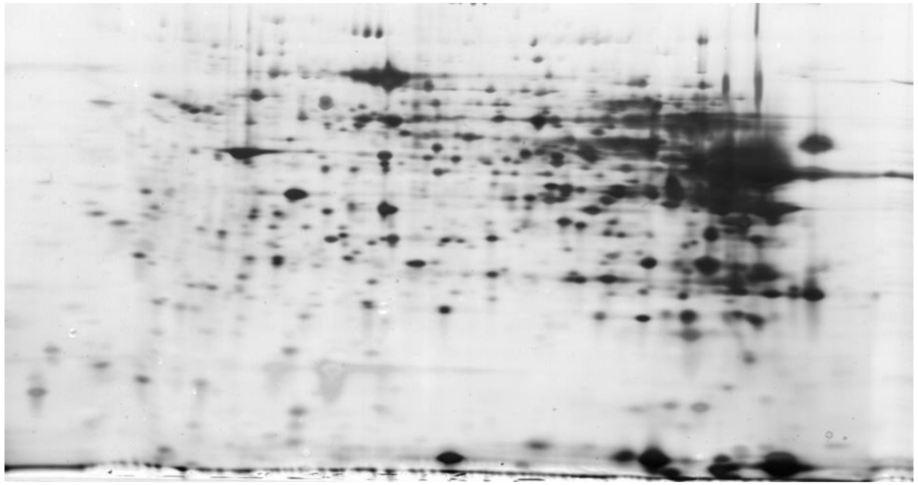

Sample 14

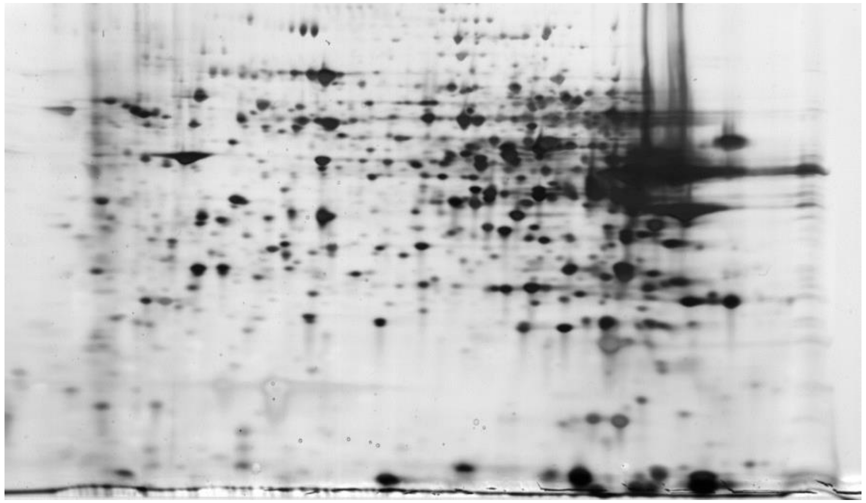

Sample 16

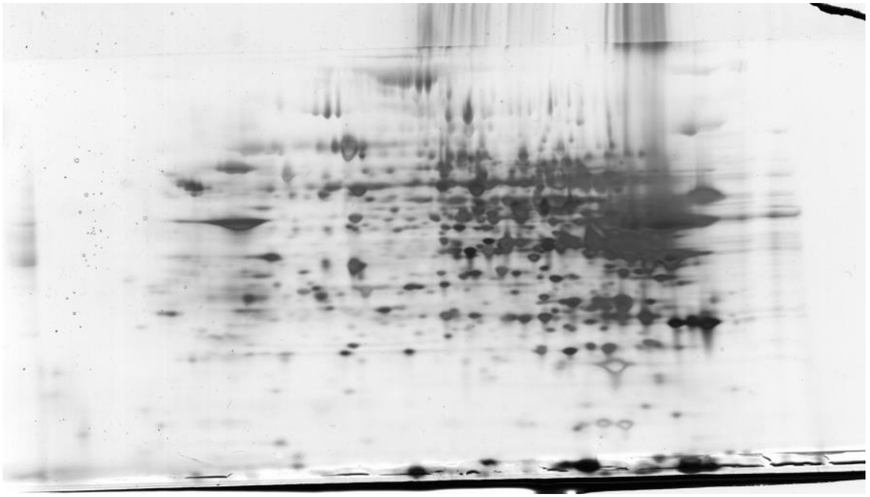

Sample 17

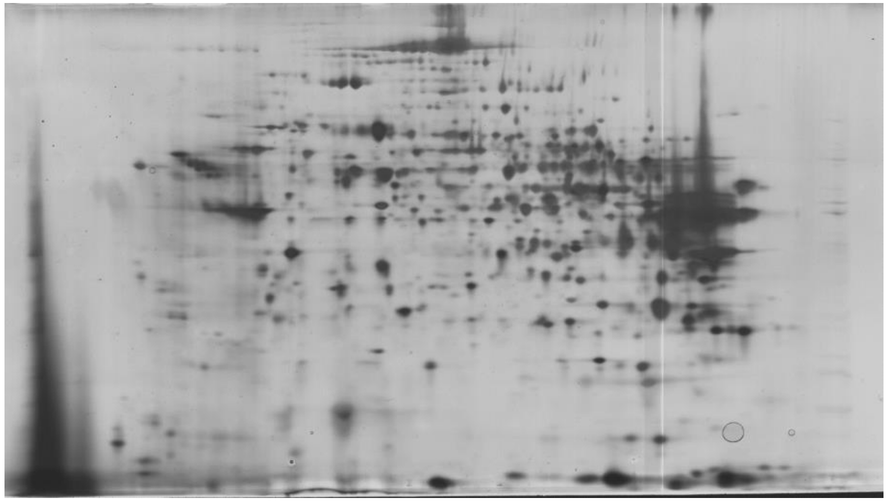

Sample 19

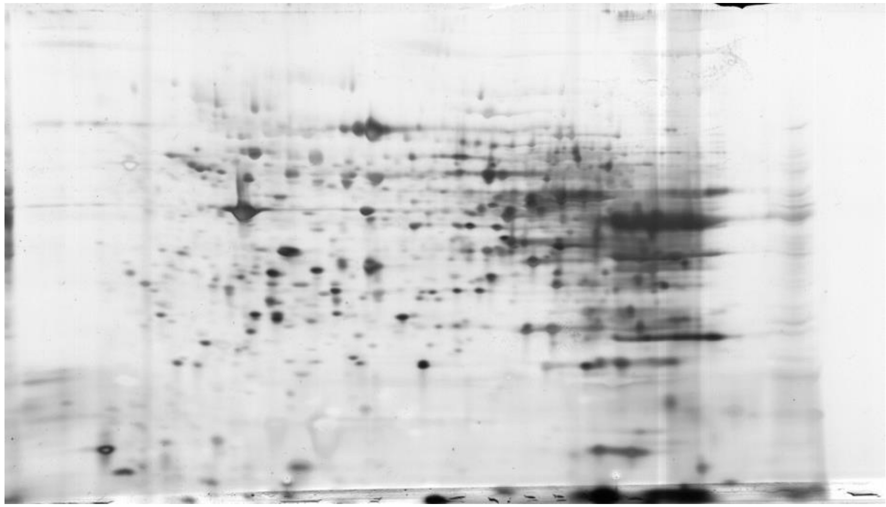

Sample 18

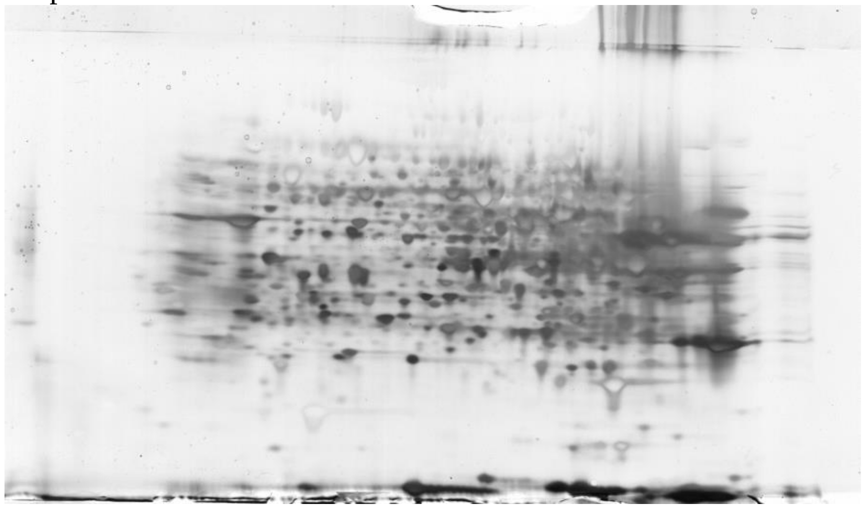

Supplement: Figure S2 — 2DE images of human liver cytosolic fraction (19 samples). 30 µg of the human soluble liver protein fraction after pretreatment with trichloroacetic acid in acetone separated by 2D-PAGE were visualized by silver staining. (PDF) [file pone.0103950.s002.pdf]
