# Supplementary material for: Applying of Hierarchical Clustering to Analysis of Protein Patterns in the Human Cancer-Associated Liver
Source: PLoS One. 2014 Aug 1;9(8):e103950. doi: 10.1371/journal.pone.0103950 (PMC4118999; doi:10.1371/journal.pone.0103950)
Supplement: Figure S3 — Mass spectrometric characterization of proteins differentially presented in cluster 1 vs cluster 2 of the human liver cytosol: a) Fructose-bisphosphate aldolase B; b) Carbamoyl-phosphate synthetase I; c) Glyceraldehyde-3-phosphate dehydrogenase; d) Glyoxylate reductase/hydroxypyruvate reductase; e) Selenium binding protein 1; and f) Superoxide dismutase [Mn], mitochondrial. (A) MALDI-TOF mass spectra of the tryptic digests of the spots on 2DE gels of human liver cytosolic fraction. Labeled peaks (*) correspond to the matched peptides of identified proteins. (B) The sequence coverage of identified protein. Matched peptides shown in bold red. (PDF) [file pone.0103950.s003.pdf]

Appendix 3: Mass spectrometric characterization of proteins differentially expressed in cluster 1 vs cluster 2 of the human liver cytosol: a) Fructose-bisphosphate aldolase B; b) Carbamoyl-phosphate synthetase I; c) Glyceraldehyde-3-phosphate dehydrogenase; d) Glyoxylate reductase/ hydroxypyruvate reductase; e) Selenium binding protein 1; and f) Superoxide dismutase [Mn], mitochondrial.

(A) MALDI-TOF mass spectra of the tryptic digests of the spots on 2D-PAGE gels of human liver cytosolic fraction. Labeled peaks (*) correspond to the matched peptides of identified proteins.

(B) The sequence coverage of identified protein. Matched peptides shown in bold red.

a) Fructose-bisphosphate aldolase B (ALDOB_HUMAN)

Swiss-Prot assession no. **P05062**

A

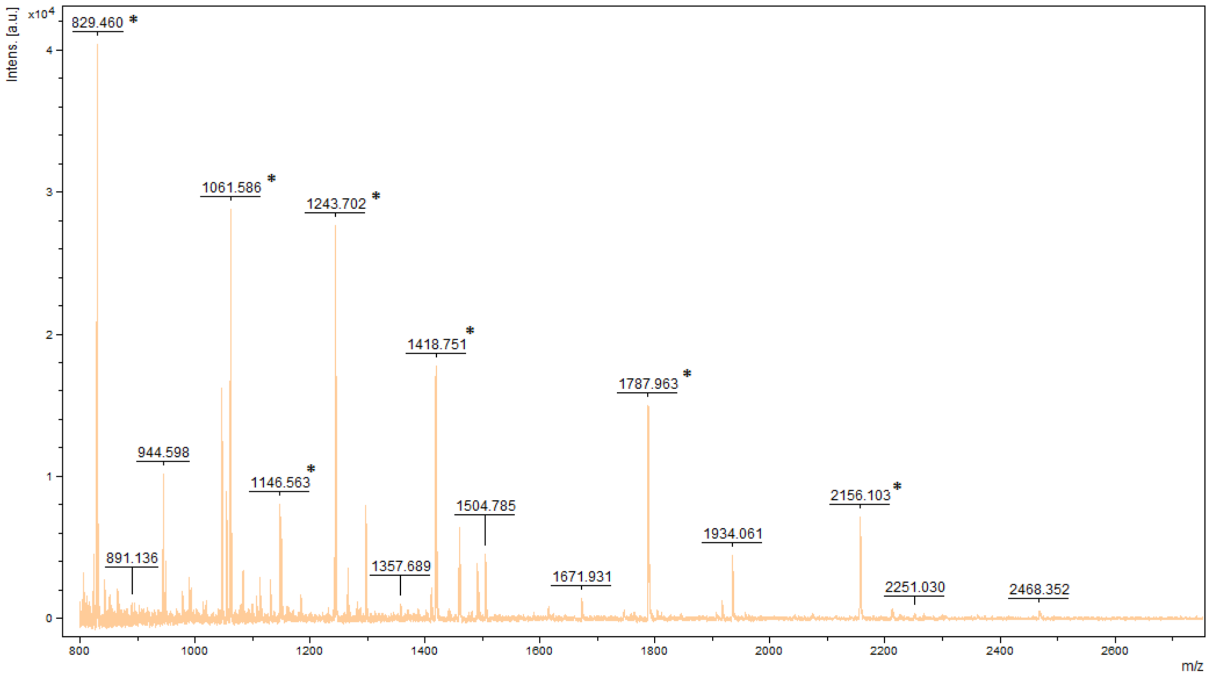

B

1 MAHRFPALTQ EQKKELSEIA QSIVANGKGI LAADESVGTM GNRLQRIKVE

51 NTEENRRQFREILFSVDSSI NQSIGGVILF HETLYQKDSQGKLFRNILKE

101 KGIVVGIKLD QGGAPLAGTN KETTIQGLDG LSERCAQYKK DGVDFGKWR

151 VLRIADQCPS SLAIQENANA LARYASICQQ NGLVPIVEPE VIPDGDHDLE

201 HCQYVTEKVL AAVYKALNDH HVYLEGTLLK PNMVTAGHAC TKKYTPEQVA

251 MATVTALHRT VPAAVPGICF LSGGMSEEDA TLNLNAINLC PLPKPWKLSF

301 SYGRALQASA LAAWGGKAAN KEATQEAFMK RAMANCQAAK GQYVHTGSSG

351 AASTQSLFTA CYTY

b) Carbamoyl-phosphate synthetase I (CPSM_HUMAN)

Swiss-Prot assession no. **P31327**

A

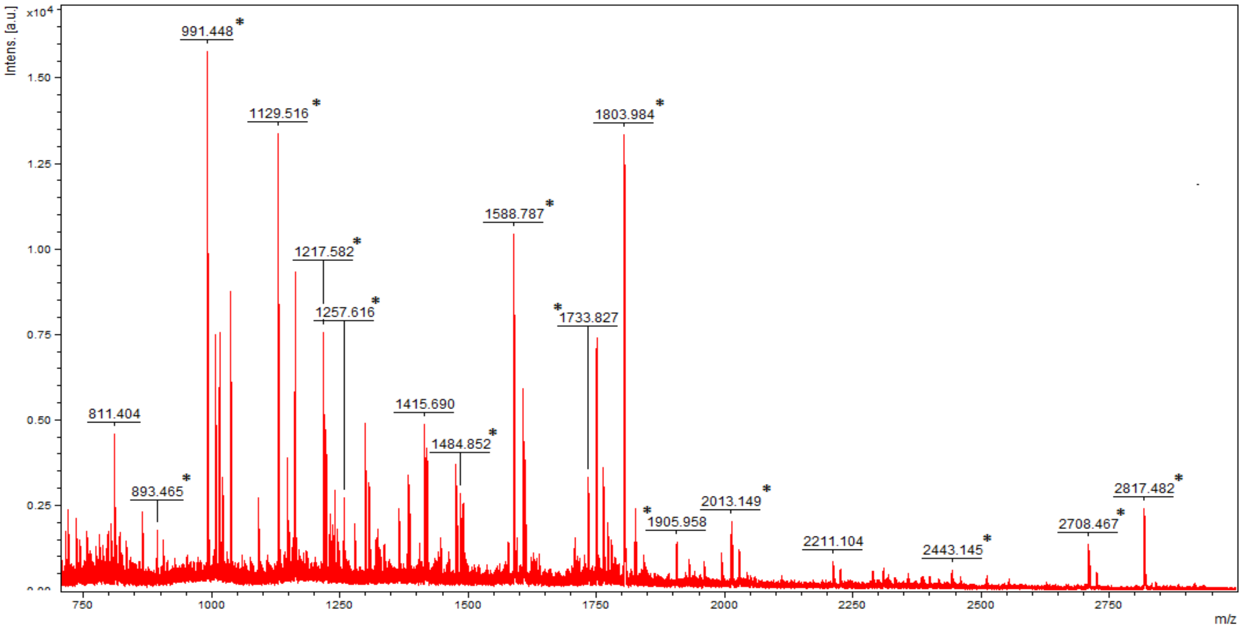

B

1	MTRILTAFKV	VRTLKTGFGF	TNVTAHQKWK	FSRPGIRLLS	VKAQTAHIVL
51	EDGTKMKGYS	FGHPSSVAGE	VVFNTGLGGY	PEAITDPAYK	GQILTMANPI
101	IGNGGAPDTT	ALDELGLSKY	LESNGIKVSG	LLVLDYSKDY	NHWLATKSLG
151	QWLQEEKVPA	IYGVDTRMLT	KIIRDKGTML	GKIEFEGQPV	DFVDPNKQNL
201	IAEVSTKDVK	VYGKGNPTKV	VAVDCGIKNN	VIRLLVKRGA	EVHLVPWNHD
251	FTKMEYDGIL	IAGGPGNPAL	AEPLIQNVRK	ILESDRKEPL	FGISTGNLIT
301	GLAAGAKTYK	MSMANRGQNQ	PVLNITNKQA	FITAQNHGYA	LDNTLPAGWK
351	PLFVNVNDQT	NEGIMHESKP	FFAVQFHPEV	TPGPIDTEYL	FDSFFSLIKK
401	GKATTITSVL	PKPALVASRV	EVSKVLILGS	GGLSIGQAGE	FDYSGSQAVK
451	AMKEENVKTV	LMNPNIASVQ	TNEVGLKQAD	TVYFLPITPQ	FVTEVIKAEQ
501	PDGLILGMGG	QTALNCGVEL	FKRGVLKEYG	VKVLGTSVES	IMATEDRQLF
551	SDKLNEINEK	IAPSFAVESI	EDALKAADTI	GYPVMIRSAY	ALGGLGSGIC
601	PNRETLMDLS	TKAFAMTNQI	LVEKSVTGWK	EIEYEVVRDA	DDNCVTVCNM
651	ENVDAMGVHT	GDSVVVAPAQ	TLSNAEFQML	RRTSINVVRH	LGIVGECNIQ
701	FALHPTSMEY	CIIEVNARLS	RSSALASKAT	GYPLAFIAAK	IALGIPLPEI
751	KNVVSGKTSA	CFEPSLDYMV	TKIPRWDLDR	FHGTSSRIGS	SMKSVGEVMA
801	IGRTFEESFQ	KALRMCHPSI	EGFTPRLPMN	KEWPSNLDLR	KELSEPSSTR
851	IYAIAKAIDD	NMSLDEIEKL	TYIDKWFLYK	MRDILNMEKT	LKGLNSESMT
901	EETLKRAKEI	GFSDKQISKC	LGLTEAQTRE	LRLKKNIHPW	VKQIDTLAAE
951	YPSVTNYLYV	TYNGQEHDVN	FDDHGMMVLG	CGPYHIGSSV	EFDWCAVSSI
1001	RTLRQLGKKT	VVVNCNPETV	STDFDECDKL	YFEELSLERI	LDIYHQEACG
1051	GCIISVGGQI	PNNLAVPLYK	NGVKIMGTSP	LQIDRAEDRS	IFSAVLDELK
1101	VAQAPWKAVN	TLNEALEFAK	SVDYPCLLRP	SYVLSGSAMN	VVFSEDEMKK
1151	FLEEATRVSQ	EHPVVLTKFV	EGAREVEMDA	VGKDGRVISH	AISEHVEDAG
1201	VHSGDATLML	PTQTISQGAI	EKVKDATRKI	AKAFAISGPF	NVQFLVKGND
1251	VLVIECNLRA	SRSFPFVSKT	LGVDFIDVAT	KVMIGENVDE	KHLPTLDHPI
1301	IPADYVAIKA	PMFSWPRLRD	ADPILRCEMA	STGEVACFGE	GIHTAFLKAM
1351	LSTGFKIPQK	GILIGIQQSF	RPRFLGVAEQ	LHNEGFKLFA	TEATSDWLNA
1401	NNVPATPVAW	PSQEGQNPSL	SSIRKLIRDG	SIDLVINLPN	NNTKFVHDNY
1451	VIRRTAVDSG	IPLLTNFQVT	KLFAEAVQKS	RKVDSKSLFH	YRQYSAGKAA

c) Glyceraldehyde-3-phosphate dehydrogenase (G3P_HUMAN)

Swiss-Prot assession no. **P04406**)

A

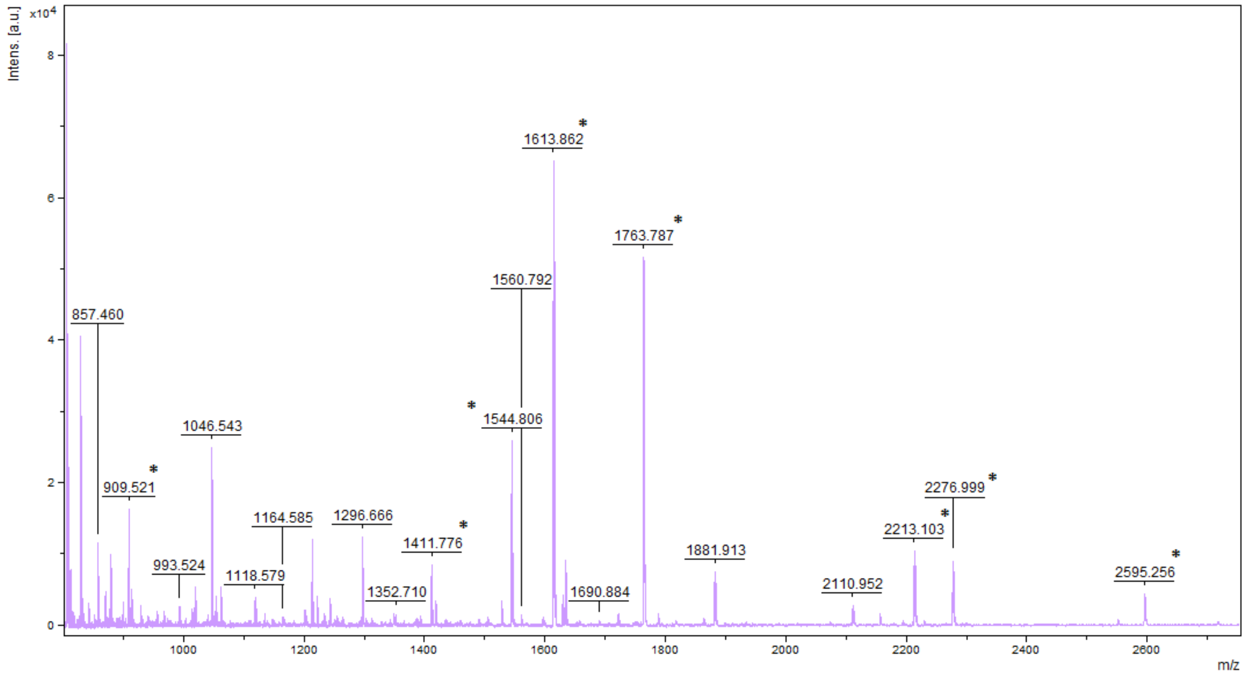

B

1 MGKVKVGVNG FGRIGRLVTR AAFNSGKVDI VAINDPFIDL NYMVYMFQYD

51 STHGKFHGTV KAENGKLVIN GNPITIFQER DPSKIKWGDA GAEYVVESTG

101 VFTTMEKAGA HLQGGAKRVI ISAPSADAPM FVMGVNHEKY DNSLKIISNA

151 SCTTNCLAPL AKVIHDNFGI VEGLMTTVHA ITATQKTVDG PSGKLWRDGR

201 GALQNIIPAS TGAAKAVGKV IPELNGKLTG MAFRVPTANV SVVDLTCRLE

251 KPAKYDDIKK VVKQASEGPL KGILGYTEHQ VVSSDFNSDT HSSTFDAGAG

301 IALNDHFVKL ISWYDNEFGY SNRVVDLMAH MASKE

d) Glyoxylate reductase/ hydroxypyruvate reductase (GRHPR_HUMAN)

Swiss-Prot assession no. **Q9UBQ7**

A

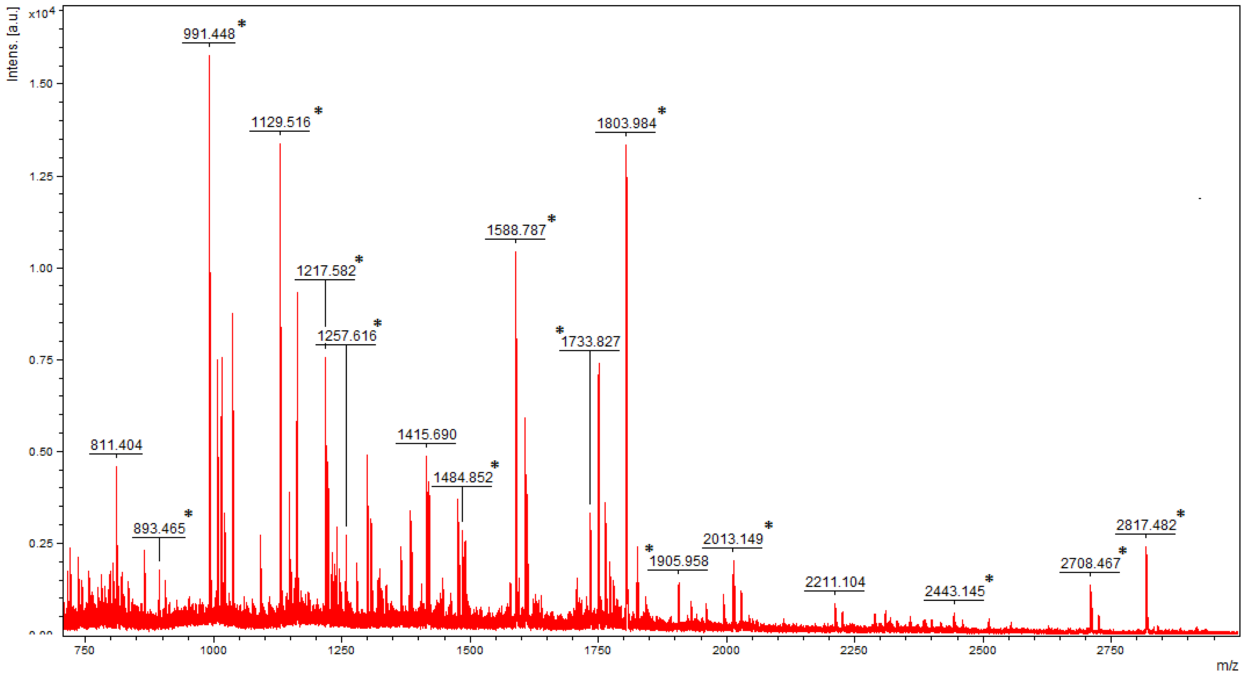

B

1 MRPVRLMKVF VTRRIPAEGR VALARAADCE VEQWDSDEPI PAKELERGVA

51 GAHGLLCLLS DHVDKRILDA AGANLKVIST MSVGIDHLAL DEIKKRGIRV

101 GYTPDVLTDT TAELAVSLLL TTCRRLPEAI EEVKNGGWTS WKPLWLCGYG

151 LTQSTVGIIG LGRIGQAIAR RLKPFGVQRF LYTGRQPRPE EAAEFQAEFV

201 STPELAAQSD FIVVACSLTP ATEGLCNKDF FQKMKETAVF INISRGDVVN

251 QDDLYQALAS GKIAAAGLDV TSPEPLPTNH PLLTLKNCVI LPHIGSATHR

301 TRNTMSLLAA NNLLAGLRGE PMPSELKL

e) Selenium binding protein 1 (SBP1_HUMAN)

Swiss-Prot assession no. **Q13228**

A

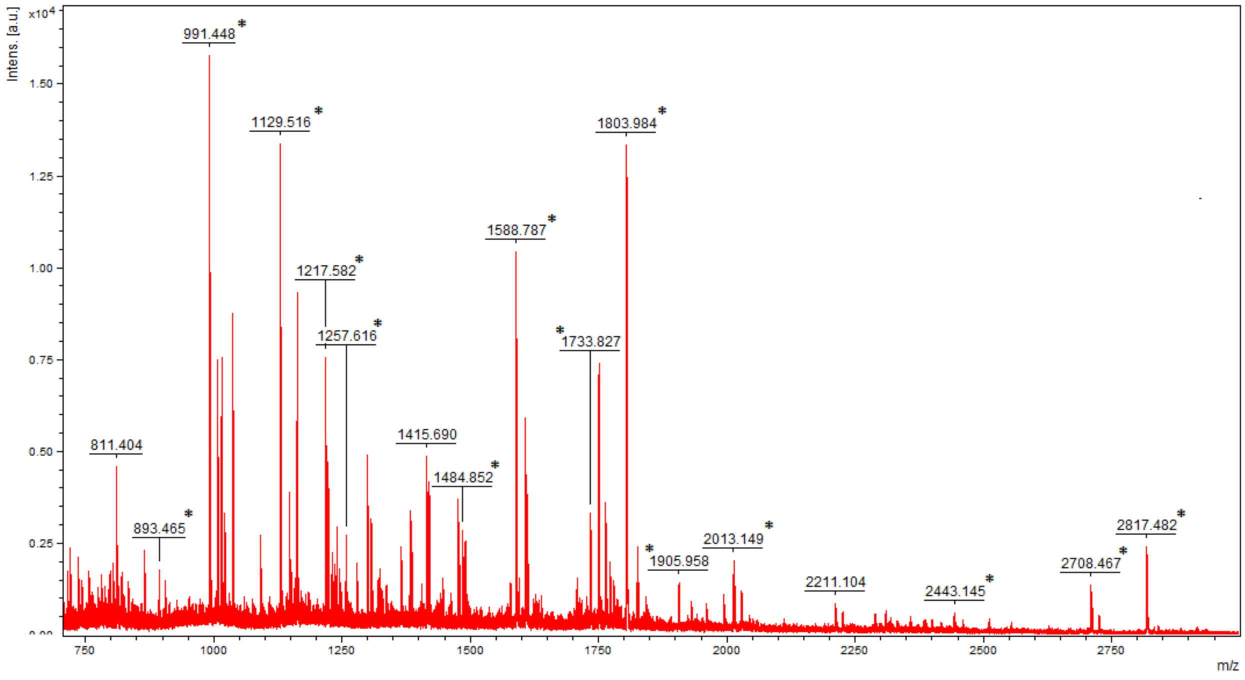

B

1 MATKCGNCGPGYSTPLEAMK GPREEIVYLP CIYRNTGTEA PDYLATVDVD

51 PKSPQYCQVI HRLPMPNLKD ELHHSGWNTC SSCFGDSTKS RTKLVLPSLI

101 SSRIYVVDVG SEPRAPKLHK VIEPKDIHAK CELAFLHTSH CLASGEVMIS

151 SLGDVKGNGK GGFVLLDGET FEVKGTWERP GGAAPLGYDF WYQPRHNVMI

201 STEWAAPNVL RDGFNPADVE AGLYGSHLYV WDWQRHEIVQ TLSLKDGLIP

251 LEIRFLHNPD AAQGFVGCAL SSTIQRFYKN EGGTWSVEKV IQVPPKKVKG

301 WLLPEMPGLI TDILLSLDDR FLYFSNWLHG DLRQYDISDP QRPRLTGQLF

351 LGGSIVKGGP VQVLEDEELK SQPEPLVVKG KRVAGGPQMI QLSLDGKRLY

401 ITTSLYSAWD KQFYPDLIRE GSVMLQVDVDTVKGGLKLNP NFLVDFGKEP

451 LGPALAHELR YPGGDCSSDI WI

f) Superoxide dismutase [Mn], mitochondrial (SODM_HUMAN)

Swiss-Prot assession no. **P04179**

A

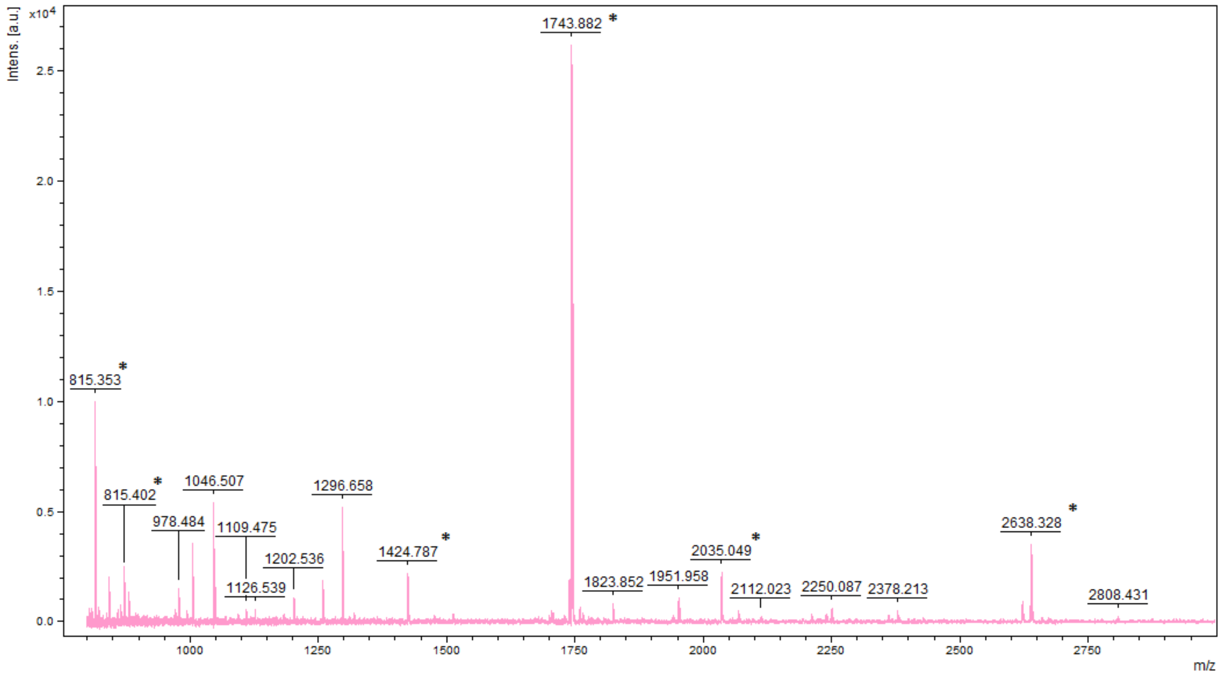

B

1 MLSRAVCGTS RQLAPALGYL GSRQKHSLPD LPYDYGALEP HINAQIMQLH

51 HSKHHAAYVN NLNVTEEKYQ EALAKGDVTA QIALQPALKF NGGGHINHSI

101 FWTNLSPNGG GEPKGELLEA IKRDFGSFDK FKEKLTAASV GVQGSGWGWL

151 GFNKERGHLQ IAACPNQDPL QGTTGLIPLL GIDVWEHAYY LQYKNVRPDY

201 LKAIWNVINW ENVTERYMAC KK
